# Scalar Relativistic All-Electron and Pseudopotential *Ab Initio* Study of a Minimal Nitrogenase [Fe(SH)_4_H]^−^ Model Employing Coupled-Cluster and Auxiliary-Field Quantum Monte Carlo Many-Body Methods

**DOI:** 10.1021/acs.jpca.3c05808

**Published:** 2024-02-07

**Authors:** Victor P. Vysotskiy, Claudia Filippi, Ulf Ryde

**Affiliations:** † Department of Computational Chemistry, Lund University, Chemical Centre, SE-221 00 Lund, Sweden; ‡ MESA+ Institute for Nanotechnology, University of Twente, P.O. Box 217, Enschede 7500 AE, Netherlands

## Abstract

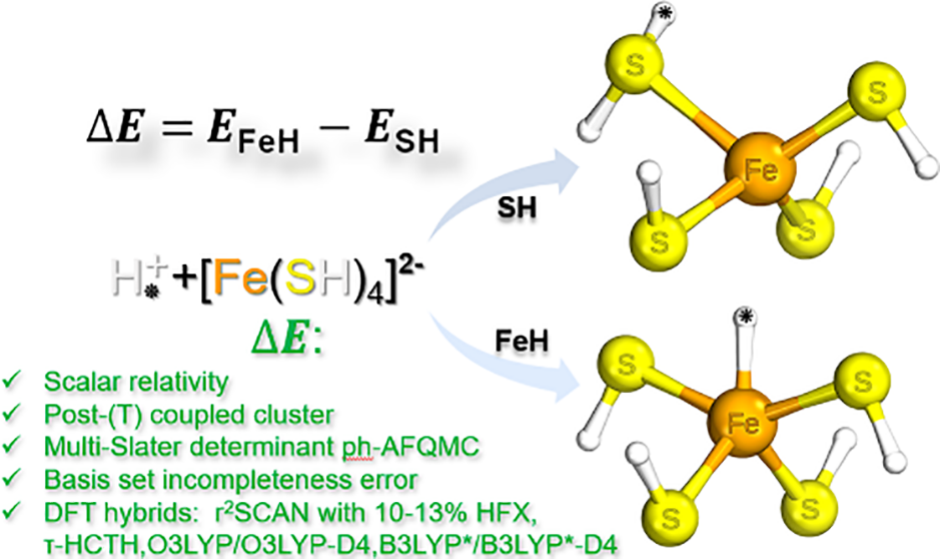

Nitrogenase is the only enzyme that can cleave the triple bond in N_2_, making nitrogen available to organisms. The detailed mechanism of this enzyme is currently not known, and computational studies are complicated by the fact that different density functional theory (DFT) methods give very different energetic results for calculations involving nitrogenase models. Recently, we designed a [Fe(SH)_4_H]^−^ model with the fifth proton binding either to Fe or S to mimic different possible protonation states of the nitrogenase active site. We showed that the energy difference between these two isomers (Δ*E*) is hard to estimate with quantum-mechanical methods. Based on nonrelativistic single-reference coupled-cluster (CC) calculations, we estimated that the Δ*E* is 101 kJ/mol. In this study, we demonstrate that scalar relativistic effects play an important role and significantly affect Δ*E*. Our best revised single-reference CC estimates for Δ*E* are 85–91 kJ/mol, including energy corrections to account for contributions beyond triples, core–valence correlation, and basis-set incompleteness error. Among coupled-cluster approaches with approximate triples, the canonical CCSD(T) exhibits the largest error for this problem. Complementary to CC, we also used phaseless auxiliary-field quantum Monte Carlo calculations (ph-AFQMC). We show that with a Hartree–Fock (HF) trial wave function, ph-AFQMC reproduces the CC results within 5 ± 1 kJ/mol. With multi-Slater-determinant (MSD) trials, the results are 82–84 ± 2 kJ/mol, indicating that multireference effects may be rather modest. Among the DFT methods tested, τ-HCTH, r^2^SCAN with 10–13% HF exchange with and without dispersion, and O3LYP/O3LYP-D4, and B3LYP*/B3LYP*-D4 generally perform the best. The r^2^SCAN12 (with 12% HF exchange) functional mimics both the best reference MSD ph-AFQMC and CC Δ*E* results within 2 kJ/mol.

## Introduction

Nitrogen is a limiting element for plant life, although the atmosphere contains 78% N_2_.^1^ The reason for this is that the triple N–N bond is strong and inert. There is only one enzyme that can cleave this bond, nitrogenase, available in a few archaea and bacteria.^1−4^ Crystallographic studies have shown that the most common isoform of this enzyme contains a complicated MoFe_7_S_9_C(homocitrate) cluster in the active site, called the FeMo cofactor.^5,6^ The enzyme catalyzes the following chemical reaction:
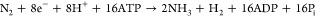
1

Thus, the enzyme consumes eight electrons and protons for each N_2_ molecule fixed. Consequently, the reaction mechanism is normally described by eight states, E_0_–E_8_, differing in the number of added electrons.^7^ It is known that H_2_ is a compulsory byproduct of the reaction and the enzyme needs to be loaded by four electrons and protons before N_2_ binds.^1,2^ ENDOR experiments have shown that E_4_ contains two hydride ions bridging two Fe ions each,^8−10^ and it is believed that H_2_ is formed by reductive elimination, leaving the FeMo cofactor in a reduced state that can bind N_2_.^8−12^ However, in spite of numerous biochemical, kinetic, spectroscopic, and computational studies, there is still no consensus regarding the detailed atomistic reaction mechanism of the enzyme.^1−4,13,14^

An important reason for this is that different density functional theory (DFT) methods give widely different energies and geometries for putative intermediates in the reaction mechanism. For example, it has been shown that the relative energy of different quadruply protonated E_4_ isomers may differ by over 600 kJ/mol when estimated by different DFT approximations.^15^ Consequently, there is an urgent need to calibrate DFT methods for models of nitrogenase. Unfortunately, there are not enough accurate and unambiguous experimental data available to calibrate the calculations, although several attempts have been made.^16−18^ Moreover, the FeMo cofactor is too large to allow for calibration with more accurate quantum mechanical (QM) methods. 3d transition-metal complexes also present a challenge to any QM method because of cooperative dynamic electronic correlation and orbital relaxation effects.^19−22^

A possible way to solve this dilemma is to use smaller models of the FeMo cofactor, which can be treated with high-level QM methods but still has relevance to the nitrogenase reaction. Recently, we developed such a model, [Fe(SH)_4_H]^−^, where the fifth proton binds either to Fe or to one of the SH groups, thereby modeling the problem of estimating the relative energies of different protonation isomers.^23^ We showed that the relative energy of the two protonation states (Δ*E*) estimated by 35 different DFT methods varied by almost 140 kJ/mol. We also estimated Δ*E* by various coupled-cluster (up to CCSDT), multiconfigurational, and semistochastic heat-bath configuration interaction methods. Our best estimate for Δ*E* was 101 kJ/mol. M06 and B3LYP were the two DFT functionals that came closest to this estimate.^23^ Meta-GGA and double hybrid DFT functionals were shown to underestimate and overestimate Δ*E* by at least 30 and 10 kJ/mol, respectively.^23^

These calculations were performed without considering any relativistic effects, which is common practice since both Fe and S are typically considered as light elements (for example, see the recent study on rubredoxin by Tzeli et al.^24^).^25^ However, incorporation of scalar relativistic effects and subvalence correlation can have a significant impact on the energetics in transition-metal complexes.^26,27^ Furthermore, the thermochemistry of transition-metal complexes is well-known to be sensitive to the description of the many-body electron correlation effects and advanced high-level *ab initio* approaches are essential to obtain accurate and reliable results.^28−33^ In particular, an appropriate level of coupled-cluster (CC) theory suitable for calibrating DFT for systems involving 3d transition metals is at the center of ongoing theoretical debates.^26,34^ Evidently, high-order CC energy corrections play an important role in transition- metal thermochemistry.^34^ In the current study, we show that relativistic effects, treated either by scalar relativistic Hamiltonians or by effective core potentials, have a significant effect on Δ*E*, lowering it by ∼12 kJ/mol. Moreover, we demonstrate that noniterative CC quadruples contributions decrease Δ*E* by 6–11 kJ/mol, and in this way, CC results approach those obtained with the phaseless auxiliary-field quantum Monte Carlo (ph-AFQMC) method. For the given model problem, ph-AFQMC with multideterminant trial wave functions provides a powerful and reliable approach to estimate Δ*E*.

## Computational Details

**Figure 1:**
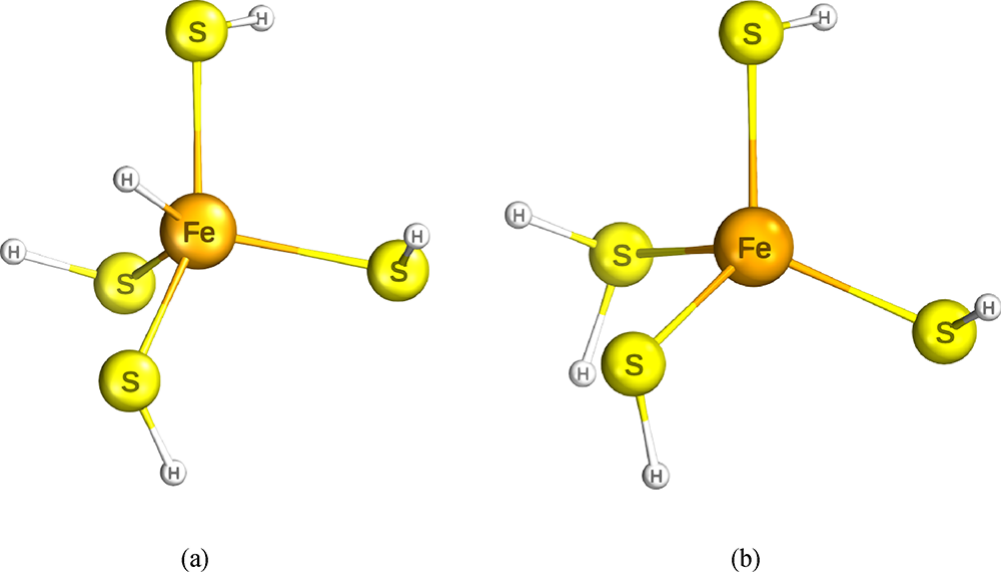
Two model systems (a) FeH and (b) SH. The Fe iron atom is colored in orange, the S sulfur atoms in yellow, and the H hydrogens in gray.

Recently, we showed that [FeH(SH)_4_]^−^ (a minimal protonated model of the active site of rubredoxin) constitutes a challenging test system for various quantum mechanical (QM) methods.^23^ In particular, the relative energy (Δ*E*) of isomers with the fifth proton bound either to one of the S atoms or to Fe is sensitive to the QM treatment, but the model is small enough so that it can be treated by high-level *ab initio* methods. The two isomers were optimized with the TPSS^35^ DFT functional and the def2-SV(P)^36^ basis set for the larger [FeH(SCH_3_)_4_]^−^ model and then truncated to [FeH(SH)_4_]^−^ using a fixed S–H distance of 1.34 Å. The models are high-spin quintet (*S* = 2) states with a net charge of −1. The total number of electrons is 96. The two structures are called FeH and SH in the following, depending on the location of the proton, and they are shown in Figure 1. The reported Δ*E* corresponds to the single-point electronic energy difference between FeH and SH for each method and basis set. Structures optimized with B3LYP,^37−39^ with the bigger def2-TZVPD basis sets,^36^ or with the X2C^40−42^ relativistic Hamiltonian are shown in Table S23. Δ*E* energy differences calculated for different structures vary by less than 3 kJ/mol.

Scalar relativistic effects can be described either explicitly, by using a one-electron relativistic Hamiltonian and a properly designed atomic basis set, or implicitly, by using effective core potentials (ECP). In the present study, we used three approaches: two explicit and one implicit. For the explicit calculations, we used two widely available scalar relativistic Hamiltonians, namely, the second-order Douglas–Kroll–Hess (DKH2) Hamiltonian^43−48^ (for CC calculations) and the spin-free one-electron exact two-component (X2C) Hamiltonian^40−42^ (for the ph-AFQMC calculations), in combination with the correlation-consistent Dunning’s Douglas-Kroll (DK) all-electron basis sets.^47,49,50^ For the implicit calculations, we used the recent correlation-consistent scalar relativistic effective core potentials (RECPs) ccECPs and corresponding basis sets,^51,52^ where the Fe and S chemical core electrons (the so-called Ne core, [1s^2^2s^2^2p^6^]) are replaced by the ECP. In the framework of ccECP, the semicore 3s and 3p electrons of Fe are treated as valence but core–valence correlation effects on the valence space are incorporated into the ECP. For comparison, we also performed nonrelativistic all-electron calculations with the corresponding polarized valence correlation-consistent Dunning’s basis sets.^49,53^

For most of the present study, within each basis set family, we used polarized valence basis sets of two sizes: either a double-ζ basis on all atoms (denoted as VDZ) or a mixed-ζ basis set, consisting of a quadruple-ζ basis set on Fe, triple-ζ on S and the reactive H atom (the one bound to Fe or S), and double-ζ on the four terminating H atoms (denoted as VXZ), which has been successfully utilized by other research studies for theoretical studies of 3d transition-metal complexes, including hydrides.^54−59^ Thus, we used three groups of basis sets, each consisting of two sizes: cc-pVDZ and cc-pVXZ,^49,53^ ccECP-pVDZ and ccECP-pVXZ,^51,52^ and cc-pVDZ-DK and cc-pVXZ-DK.^47,49,50^ In the all-electron case, the total number of basis functions is 140 and 274 for the double-ζ and mixed-ζ basis sets, respectively. Despite the fact that, for a given ζ-contraction scheme, the total number of basis functions remains the same and similar naming convention is used for the basis-set families, the basis sets differ in terms of primitive exponents and/or contractions coefficients. For instance, the primitive Gaussian functions are equal but the contraction coefficients are different between the cc-pVDZ (cc-pVXZ) and cc-pVDZ-DK (cc-pVXZ-DK) basis sets, whereas the original ccECP basis sets have their own unique primitive Gaussian functions and contraction coefficients.

To estimate the basis-set incompleteness error (BSIE) at the CC level of theory, we used standard cc-pV*Y*Z-DK and ccECP-pV*Y*Z atomic basis sets, where *Y* = *T*, *Q*, and 5, on Fe, S, and the reactive H, while always keeping the corresponding double-ζ quality basis set on the other four terminating H. To investigate inner core–valence correlation effects, we additionally augmented the mixed-ζ Dunning’s DK basis set on Fe and S by using basis functions with tight exponents, namely, the cc-pwCVQZ-DK^60^ and cc-pCVTZ-DK^61^ basis sets on Fe and S, respectively. For the sake of clarity, this extended correlation-consistent polarized core–valence basis set is hereinafter referred to as the cc-pCVXZ-DK. Detailed basis set specifications are given in Table S1 in the Supporting Information.

The ph-AFQMC calculations with single-determinant and multi-Slater determinant trial wave functions were performed with the IPIE^62^ and DICE^63,64^ packages, respectively. To prevent spin-contamination, all trial wave functions were originally optimized using PySCF^65^ and the restricted open-shell Hartree–Fock (ROHF)^66^ and Kohn–Sham (ROKS)^67,68^ methods. The ROHF and ROKS reference wave functions with a cc-pVXZ-DK basis set were generated using the X2C Hamiltonian. The HFLYP^38,69^ exchange–correlation (XC) functional was used solely to produce a single-determinant trial wave function for subsequent ph-AFQMC calculations.

The ph-AFQMC propagation utilized an imaginary time step of 0.005 au (sometimes referred to *E*_*h*_^–1^ or β in the literature). Due to the different computer architectures (in terms of CPU cores available per compute node), we used from 1920 to 2048 walkers for the calculations. To reach a consistent Δ*E* with a similar level of stochastic noise for a given combination of basis set and trial wave functions, the number of walkers was always kept equal for the two isomers. The Cholesky factorization necessary for single- and multideterminant ph-AFQMC calculations was performed with tight Cholesky thresholds of 10^–7^ and 5 × 10^–6^, respectively. Unless stated otherwise, the total number of propagated blocks were 10000 and 4500 for single- and multideterminant trials, respectively. Each block consisted of 25 propagation steps and one energy evaluation and orthogonalization. Throughout the calculations, the equilibration time was set to 2.0 au Population control, and local energy measurements were carried out every 5 steps.

Spin-restricted open-shell similarity-transformed CCSD(2),^70^ locally renormalized LR-CCSD(T), IIIB^71,72^ (henceforth denoted as LR-CCSD(T)) and LR-CCSD(TQ)-1^71,72^ energies were computed in NWChem.^73−77^ Spin-restricted open-shell coupled-cluster (CC) calculations with singles and doubles (CCSD),^78^ rigorously size-extensive completely renormalized CR-CC(2,3),^79,80^ and CC(t;3)^81,82^ were run with GAMESS (US) 2021R2.^83^ In the case of CC(t;3), the four singly occupied α and the corresponding four unoccupied β molecular spin orbitals formed the active space for full treatment of the triples energy correction, i.e., the default settings in GAMESS (US). In the spin-restricted open-shell CC calculations, the *h*-type functions of the ccECP-pVXZ and cc-pVXZ-DK basis sets on Fe were omitted because the GAMESS (US) integral code can only handle up to *g*-type basis functions. All-electron scalar relativistic open-shell spin-restricted calculations were performed using the DKH2 Hamiltonian. Both nonrelativistic and DKH2 scalar relativistic all-electron calculations at the spin-unrestricted open-shell CCSD(T) and Brueckner coupled-cluster doubles with perturbative triples (UBCCD(T))^84,85^ levels used the PSI4^86^ package. Scalar relativistic X2C and ECP UBCCD(T) calculations were performed using the PySCF^65^ quantum chemistry package. The all-electron spin-unrestricted UCC3^87^ calculations were performed with the PSI4 software.

Unrestricted Kohn–Sham DFT calculations were performed using the Gaussian 16 package,^88^ but those involving the r^2^SCAN^89,90^ and HFLYP^38,69^ XC functionals were run with NWChem^76,77^ and PySCF,^65^ respectively. For all DFT calculations, we used the finest integration grid available. We used nine well-established DFT XC functionals: TPSSh,^91^ r^2^SCANh,^92,93^ r^2^SCAN0,^92,94^ B3LYP,^37−39^ B3LYP*,^95,96^ O3LYP,^97,98^ τ-HCTH,^99^ M05,^100^ and M06.^101^ Empirical D4^92,102−104^ and nonlocal electronic density-based VV10^105^ dispersion energy corrections were computed either directly in ORCA^106^ or by using the external DFT-D4 program by Grimme et al.^107^ All reported VV10 energy corrections were computed non-self-consistently. The VV10 corrections for the r^2^SCAN-based DFT XC functionals were calculated with the global parameter *b* = 11.95.^108^

All post-Hartree–Fock wave function-theory-based all-electron calculations with polarized valence Dunning’s basis sets were performed using the frozen-core approximation, i.e., the 1s, 2s, and 2p inner-shell electrons were kept frozen in S and Fe (these are the same electrons treated by the ECP). Therefore, only 46 electrons were treated as active (25 α and 21 β). However, CC calculations with the polarized core–valence cc-pCVXZ-DK basis set were performed with fully correlated 2s and 2p subvalence electrons in both S and Fe, i.e., only 10 electrons were kept frozen.

For the complete basis-set-limit (CBS) extrapolation, we employed the two-parameter inverse cubic extrapolation scheme,^109,110^ the mixed Gaussian/exponential extrapolation scheme,^111^ the modified inverse cubic two-point CBS extrapolation scheme,^112^ and the Riemann zeta function approach.^113^ In the latter case, the unified single-parameter extrapolation scheme by Varandas et al. was used to extrapolate Hartree–Fock energies individually.^114,115^

### Canonical Coupled-Cluster Results

The coupled-cluster method with singles, doubles, and perturbative triples (CCSD(T)) is often referred to as the “gold standard” of computational chemistry because of its high accuracy and feasible computational scaling, *O*(*N*^7^), where *N* is the number of basis functions. For the present model using the nonrelativistic Dunning’s cc-pVDZ basis set, we recently showed that canonical CCSD(T) calculations with Hartree–Fock (HF) reference orbitals exhibit a rather large error for Δ*E* (>7 kJ/mol), while BCCD(T) provides an excellent accuracy (0.5 kJ/mol), compared to full CCSDT calculations.^23^ Furthermore, the BCCD(T) method consistently approaches CCSDT for both the total energy of the model structures and Δ*E*. It was also demonstrated that the results of the CR-CC(2,3) and UBCCD(T) methods agree within 1 kcal/mol (4.184 kJ/mol), i.e., within so-called “chemical accuracy”. Since CCSDT calculations become prohibitively expensive when utilizing the mixed high-quality VXZ basis sets, we employ in this work the advanced LR-CCSD(T) and CC(t;3) coupled-cluster approaches to retain near-CCSDT-quality energies with high accuracy and to complement UBCCD(T). Having an independent set of high-level energies is important, especially in situations where a covalent bond is forming or breaking and thereby the perturbative energy correction might fail.^116,117^

In practice, due to the severe high-degree polynomial or even exponential scaling of advanced *ab initio* methods, double-ζ quality compact basis sets (e.g., def2-SVP or cc-pVDZ) are still in wide use to calibrate the general performance of novel many-body methods. To examine the capability of the rigorous LR-CCSD(T) and CC(t;3) approaches, we first computed both the absolute and relative nonrelativistic energies using the cc-pVDZ basis set. The CC(t;3)/cc-pVDZ and LR-CCSD(T)/cc-pVDZ results are given in Table 1 and are compared to the UBCCD(T), CR-CC(2,3), and UCCSDT energies from ref (23).

**Table 1:** Nonrelativistic CC Total (au) and Relative Δ*E* (kJ/mol) Energies Computed Using the cc-pVDZ Basis Set

	total energy (au)				
structure	CR-CC(2,3)^23^	CC(t;3)	UBCCD(T)^23^	LR-CCSD(T)	UCCSDT^23^
FeH	–2856.685849	–2856.687346	–2856.687449	–2856.690398	–2856.689019
SH	–2856.726088	–2856.726560	–2856.726121	–2856.726143	–2856.727886

	Δ*E* (kJ/mol)				
	CR-CC(2,3)^23^	CC(t;3)	UBCCD(T)^23^	LR-CCSD(T)	UCCSDT^23^
	105.7	103.0	101.5	93.8	102.0

As can be seen from Table 1, the CC(t;3) and UBCCD(T) results closely agree, despite the vastly different underlying reference Hartree–Fock wave functions and formalisms. Specifically, the total electronic energies agree within 1.2 kJ/mol, and the two methods differ by only 1.5 kJ/mol in Δ*E*. Compared to the most accurate standalone UCCSDT result, CC(t;3) overestimates Δ*E* by 1.0 kJ/mol, whereas UBCCD(T) underestimates Δ*E* by 0.5 kJ/mol. Thus, both the CC(t;3) and UBCCD(T) methods agree consistently and quantitively with UCCSDT. Among the noniterative triples corrections to CCSD, the LR-CCSD(T) method predicts an appreciably lower Δ*E* value of 93.8 kJ/mol, and the main source of the difference in Δ*E* is undoubtedly the energy of the FeH structure. Relative to UCCSDT, all perturbative triples CC methods underestimate the total electronic energies of SH by ∼1.3–1.8 millihartree (mE_h_; 3–5 kJ/mol), but LR-CCSD(T) gives the lowest electronic energy for FeH (∼1 mE_h_ lower than the UCCSDT results, whereas the other methods overestimate also this energy).

**Figure 2:**
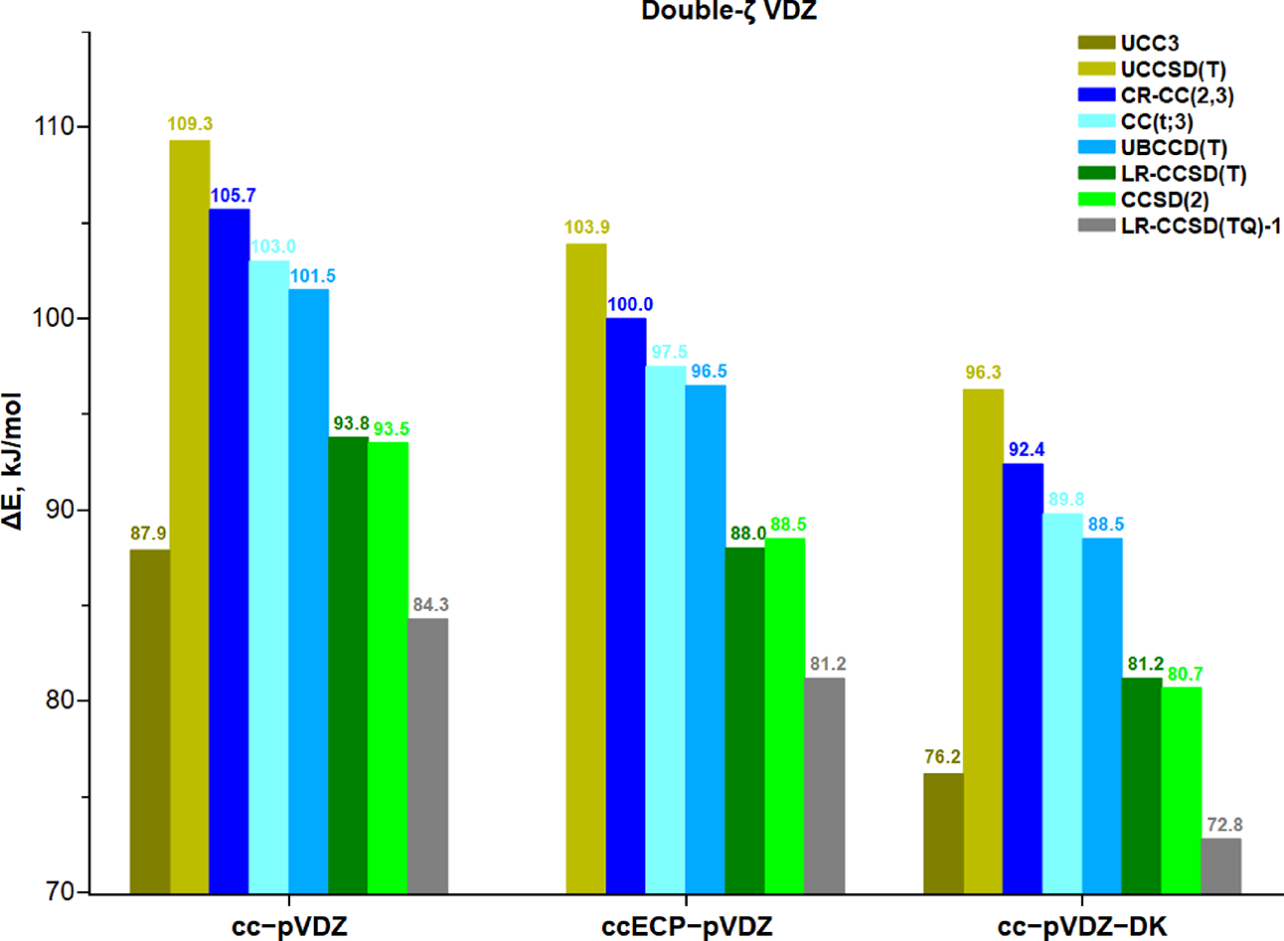
Energy difference between the two protonated model structures (Δ*E* in kJ/mol) computed with various CC approaches, three Hamiltonians, and the double-ζ basis sets. The nonrelativistic UCCSD(T)/cc-pVDZ and UCC3/cc-pVDZ results are taken from ref (23) .

Needless to say, the contribution of higher-order CC energy corrections (HOC) beyond (perturbational) triples may be small in absolute terms but may be chemically quite significant in relative terms. Unfortunately, an iterative CCSDT(Q) approach with perturbative quadruples is computationally prohibitive for the studied model, and only the perturbative triple and quadruple CCSD(2) and LR-CCSD(TQ)-1 methods (TQ) are feasible on our available hardware with double-ζ basis sets. The relative Δ*E* energies of the different perturbative triples and quadruples approaches are depicted in Figure 2.

To highlight a potential breakdown of novel CC approaches, the results of the ubiquitous UCCSD(T) and of the iterative triple UCC3 method are also shown in Figure 2. With the all-electron double-ζ quality Dunning’s basis sets, the nonrelativistic and scalar relativistic UCC3 results are always in between the high-order CCSD(2) and LR-CCSD(TQ)-1 results. However, it is unclear whether good error compensation or proper treatment of singles amplitudes without any approximations and/or relaxation of perturbative triples in the presence of singles and doubles brings CC3 closer to the high-order (TQ) methods, an outstanding accuracy for which we have no convincing explanation at this time. UCCSD(T) is seen to give the worst performance. For this reason, we decided to exclude CCSD(T) from any further discussion. Furthermore, with our hardware and software, the iterative triples UCC3 calculations are intractable with mixed-ζ as well as ECP basis sets, and no further calculations were carried out with this method.

For all double-ζ basis sets, with and without scalar relativistic Hamiltonians, the other six CC approaches cluster into five groups as seen in Figure 2: the maximal and minimal estimates on Δ*E* are given by CR-CC(2,3) and LR-CCSD(TQ)-1 methods, respectively; CC(t;3) and UBCCD(T) form a pair of results, while LR-CCSD(T) compares favorably with the higher-order CCSD(2) method; UCC3 is in between CCSD(2) and LR-CCSD(TQ)-1. Regarding the perturbative (TQ) approaches, an inclusion of post-triples HOC substantially reduces Δ*E*. On average, CCSD(2) and LR-CCSD(TQ)-1 predict a lower Δ*E* by 8 and 17 kJ/mol compared to those obtained with CC(t;3) or UBCCD(T). In either case, a general trend of decreasing Δ*E* going toward more advanced CC approaches is quite apparent, and therefore, HOC cannot be neglected and must be somewhat taken into account at the CC level. We will suggest a pragmatic solution to this problem in the subsequent section.

**Figure 3:**
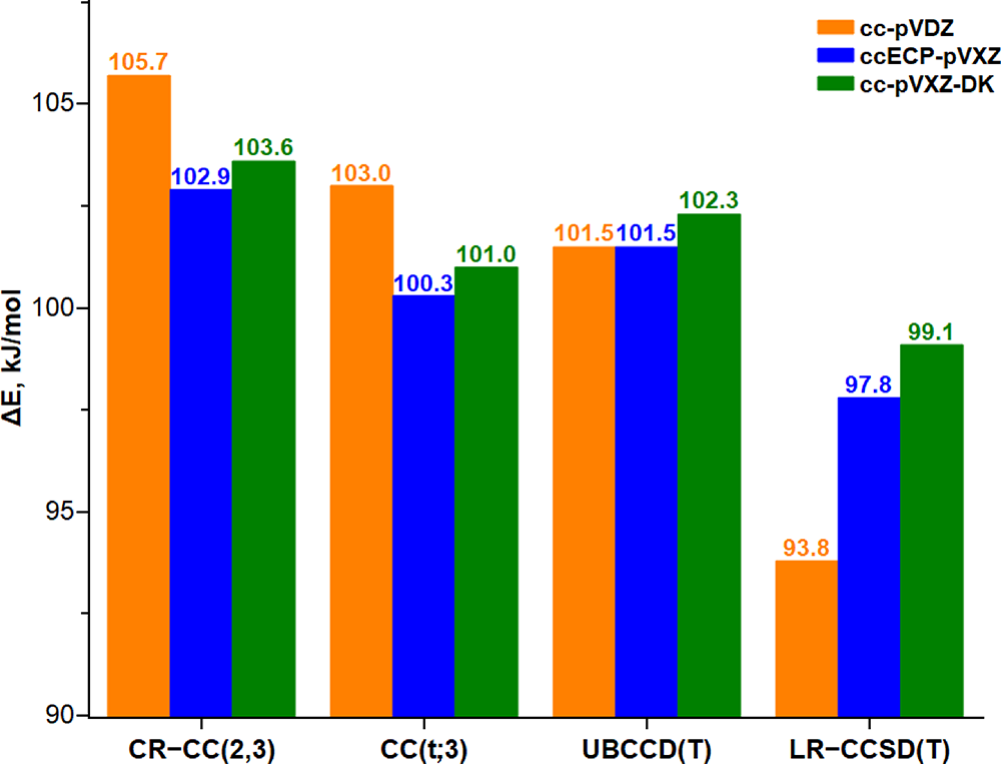
Energy difference between the two protonated model structures (Δ*E* in kJ/mol) computed with the various CC approaches, the three Hamiltonians, and the corresponding basis sets, viz., all-electron nonrelativistic double-ζ cc-pVDZ, mixed-ζ correlation-consistent effective core-potential ccECP-pVXZ, and mixed-ζ all-electron scalar relativistic cc-pVXZ-DK.

In Table 2, we show relativistic and nonrelativistic CR-CC(2,3), CC(t;3), UBCCD(T), and LR-CCSD(T) results for the six basis sets and Hamiltonians. It can be seen that there are two distinct and opposite trends: the inclusion of scalar relativistic effects, either directly via DKH2 or indirectly via ECP, always decreases Δ*E*, whereas improving the quality of the atomic basis sets increases Δ*E*. In particular, Δ*E* becomes 12–14 kJ/mol smaller with the scalar relativistic Hamiltonian and the DK basis sets regardless of their size. For the double-ζ quality VDZ basis sets, the CC/ccECP results are always in between the all-electron scalar relativistic and nonrelativistic results, ∼8 kJ/mol larger than the former. However, with the larger mixed-ζ quality VXZ basis sets, the CC/ccECP-pVXZ and all–electron CC/cc-pVXZ-DK results get much closer and agree within 1.3 kJ/mol (ECP results are always smaller). On the other hand, for the all-electron nonrelativistic calculations, enlarging the basis set from VDZ to VXZ increases Δ*E* by 11–18 kJ/mol, with the higher value corresponding to LR-CCSD(T). In total, the two opposite trends effectively compensate each other, and thereby, the nonrelativistic calculations with the cc-pVDZ basis set give fortuitously results that are in reasonable agreement (within 3–5 kJ/mol) with the relativistic ccECP and DK results with the VXZ basis set, as can be seen in Figure 3.

**Table 2:** Energy Difference Δ*E* Computed by Employing Various CC Approaches and Basis Sets (in kJ/mol)

		method			
Hamiltonian	basis set	CR-CC(2,3)	CC(t;3)	UBCCD(T)	LR-CCSD(T)
nonrelativistic	cc-pVDZ	105.7	103.0	101.5	93.8
RECP	ccECP-pVDZ	100.0	97.5	95.6	88.0
DKH2	cc-pVDZ-DK	92.4	89.8	88.5	81.2
nonrelativistic	cc-pVXZ	117.7	114.9	114.9	111.5
RECP	ccECP-pVXZ	102.9	100.3	101.5	97.8
DKH2	cc-pVXZ-DK	103.6	101.0	102.3	99.1

To further understand the impact of relativity on Δ*E*, we decompose Δ*E* into a mean-field HF and a many-body CC contribution for the mixed-ζ quality VXZ basis sets, as shown in Table 3. Clearly, scalar relativity mainly affects Δ*E* at the HF level of theory, decreasing it by 15–19 and 20–22 kJ/mol at the ROHF and UHF levels of theory, respectively. Thus, including scalar relativity induces core–valence polarization effects, which are accounted for already at the HF level. Other many-body correlation effects between inner-core and semicore-plus-valence electrons (CV) were estimated via CC calculations with a small core (10 electrons) and appropriate polarized core–valence basis sets. As shown in Table S6 in the Supporting Information, such CV effects have only a slight impact on Δ*E* and cause an increase by about 1 kJ/mol only. More specifically, the CV energy corrections are 1.3 and 1.2 kJ/mol for the UBCCD(T) and LR-CCSD(T) methods, respectively. This CV energy correction of 1.3 kJ/mol will be applied later to correct the all-electron results computed using Dunning’s DK basis sets and the frozen-core approximation.

**Table 3:** Energy Difference Δ*E* Decomposed into Mean-Field HF and Many-Body CC Contributions, Computed Employing Various CC Approaches, Hamiltonians, and Mixed-ζ Quality VXZ Basis Sets (in kJ/mol)

		method				
Hamiltonian	basis set	ROHF	CC(t;3)	LR-CCSD(T)	UHF	UBCCD(T)
nonrelativistic	cc-pVXZ	397.7	–282.7	–286.2	268.7	–153.8
RECP	ccECP-pVXZ	382.3	–282.0	–284.5	249.0	–147.4
DKH2	cc-pVXZ-DK	378.6	–277.6	–279.5	246.3	–144.1

All raw CC energies for both structures discussed in this section are available in Tables S2 and S6 in the Supporting Information.

### Coupled-Cluster Results Extrapolated to the FCI Limit

The electronic energies computed using the truncated CCSDT expansion can be further extrapolated toward the full configuration interaction (FCI) limit by using the quadratic Padé approximant:^118,119^
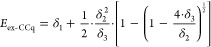
2where δ_1_ = *E*_HF_, δ_2_ = *E*_CCSD_ – *E*_HF_, and δ_3_ = *E*_CCSDT_ – *E*_CCSD_.

In this way, one can account for the missing higher-order excitations and obtain corrected total electronic energies that approach the corresponding FCI exact result (for a given basis set).^120,121^ Recently, we demonstrated that such an extrapolation is essential to put on equal footing standalone CC and the near-exact semistochastic heat-bath configuration interaction (SHCI) energies for nonrelativistic calculations on the same model systems.^23^ Specifically, the total and relative energies of extrapolated CC and SHCI were within 5 kJ/mol.

Generally speaking, the quadratic Padé approximant can be applied to any sequence of CC energies. In practice, for medium-sized molecules and large basis sets, the full CCSDT calculations are unfeasible due to the *O*(*N*^8^) scaling, and only approximate triple-excitation CC approaches are affordable. In the present work, for the case of perturbative-triples corrected CCSD methods, we computed the δ_1_–δ_3_ terms based on the ROHF – ROCCSD – CC(t;3), ROHF – ROCCSD – LR-CCSD(T), and UHF – UCCSD – UBCCD(T) energies. Since we have computed both LR-CCSD(T) and LR-CCSD(TQ)-1 energies using double-ζ quality VDZ basis sets, it is natural to use the ROCCSD – LR-CCSD(T) – LR-CCSD(TQ)-1 extrapolation. The CCSD(2) energies were extrapolated using the ROHF – ROCCSD – CCSD(2) results since CCSD(2) is formulated as a true second-order correction to CCSD.^70^ Henceforth, we denote the extrapolated results ex-UBCCD(T), ex-LR-CCSD(T), ex-CC(t;3), ex-CCSD(2), and ex-LR-CCSD(TQ)-1 to distinguish the ex-CCq energies resulting from the use of eq 2. The CR-CC(2,3) method was excluded from extrapolation because it is the least accurate among the novel CC approaches used.

**Figure 4:**
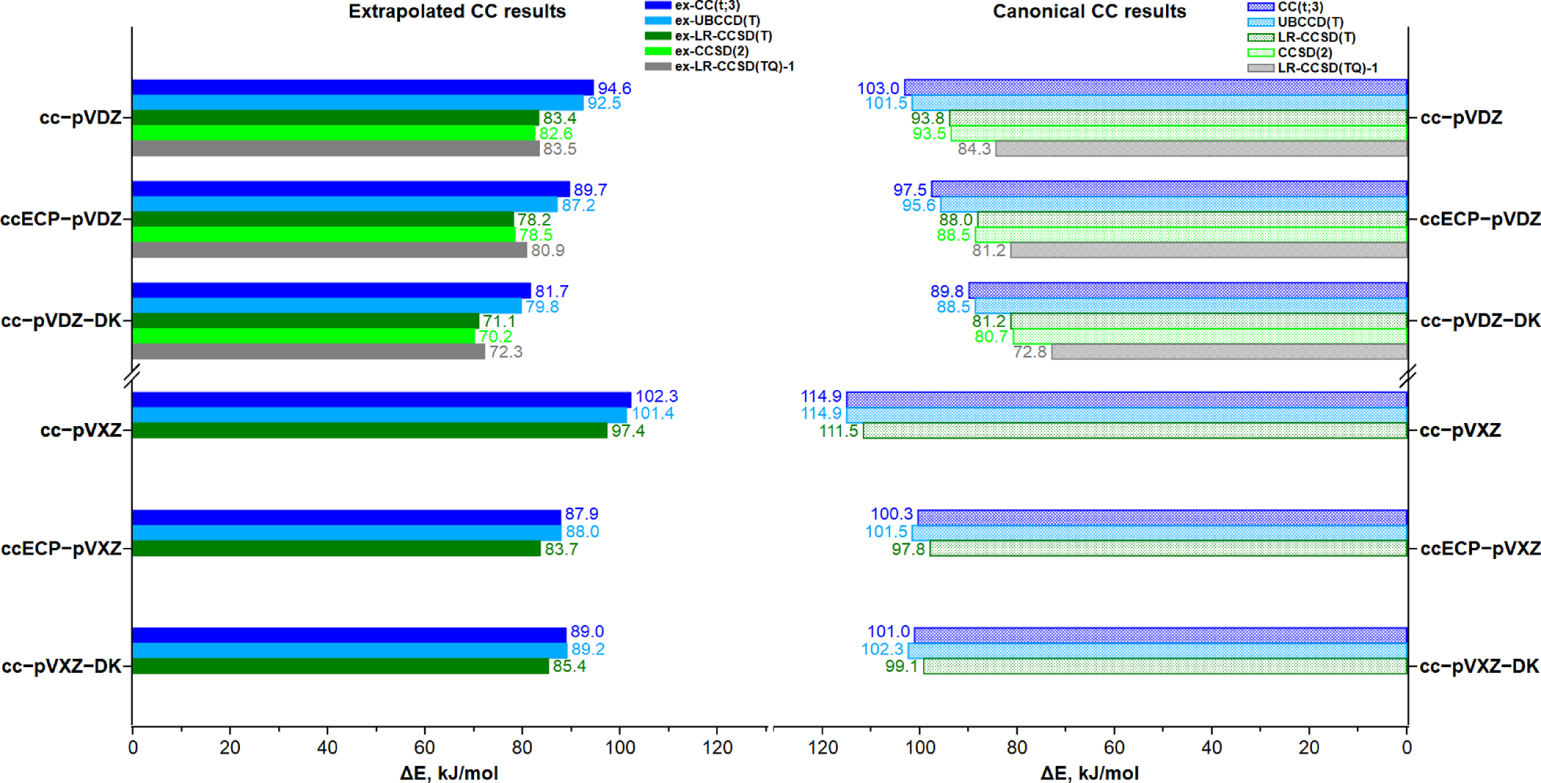
Energy difference Δ*E* (kJ/mol) between the two protonated model structures computed with various CC approaches and Hamiltonians/basis sets. The extrapolated and canonical CC results are shown on the left- and right-hand sides, respectively.

In Figure 4, Δ*E* computed from the CC results extrapolated to the FCI limit is shown along with the corresponding standalone CC results. Overall, all trends observed for the canonical CC results remain the same. In particular, the extrapolated ex-UBCCD(T) and ex-CC(t;3) Δ*E* results agree fairly well: the largest difference encountered is 2.5 kJ/mol with the VDZ basis sets and 1.0 kJ/mol with the VXZ basis sets. Furthermore, Δ*E* calculated with LR-CCSD(T) agrees with the higher-order CCSD(2) value within 1 kcal/mol, regardless of the Hamiltonian and basis set. Finally, the most fascinating result is that the extrapolated high-order (TQ) ex-CCSD(2) and ex-LR-CCSD(TQ)-1 Δ*E* values display very good agreement within 2.4 kJ/mol.

When the extrapolated CC Δ*E* results are compared to the corresponding standalone ones, it is evident that the Padé extrapolation uniformly decreases Δ*E* by 8–10 and 12–14 kJ/mol for double- and mixed-ζ quality basis sets, respectively. The only exception is the LR-CCSD(TQ)-1 result, which remains essentially unchanged (<0.8 kJ/mol) upon the Padé extrapolation. Taking into account the invariance of the LR-CCSD(TQ)-1/VDZ Δ*E* result with respect to the extrapolation with the Padé extrapolation, we decided to compute additive HOC energy corrections for each perturbative triples CC method relative to the LR-CCSD(TQ)-1/VDZ results. For each combination of basis set family and CC method, the resulting HOC corrections are given in Table 4.

**Table 4:** High-Order (TQ) A Posteriori Additive Energy Corrections for the Extrapolated CC Δ*E* Energies (in kJ/mol)

		HOC		
Hamiltonian	basis set family	ex-CC(t;3)	ex-UBCCD(T)	ex-LR-CCSD(T)
nonrelativistic	Dunning’s	–11.1	–9.0	–0.1
RECP	ccECP	–8.8	–6.3	2.7
DKH2	Dunning’s DK	–9.4	–7.5	1.0

We should emphasize that the HOC correction for ex-LR-CCSD(T) is rather small for both scalar relativistic and nonrelativistic Dunning’s basis sets. In other words, with the all-electron Dunning’s basis sets, the HOC corrected ex-LR-CCSD(T) Δ*E* misses in practice quadruple corrections and rather represents the canonical perturbative triples result only. On the other hand, in the case of the CC(t;3) and UBCCD(T) approaches, the HOC (TQ) CC energy correction to Δ*E* might have a smaller impact with larger than double-ζ basis sets.^122^ Therefore, one can speculate that, with the mixed-ζ VXZ Dunning’s basis sets, the extrapolated CC estimates of Δ*E* set a lower limit in the case of ex-CC(t;3)-HOC and ex-UBCCD(T)-HOC, and an upper limit in the case of ex-LR-CCSD(T)-HOC.

For the scalar relativistic cc-pVXZ-DK and ccECP-pVXZ basis sets, the extrapolation and a posteriori HOC correction further reduce Δ*E* to 79, 82, and 86 kJ/mol for CC(t;3), UBCCD(T), and LR-CCSD(T), respectively, with a maximal deviation of only 0.5 kJ/mol between the two relativistic calculations for each CC method. Hereinafter, these results will be referred to as ex-CC(t;3)-HOC, ex-UBCCD(T)-HOC, and ex-LR-CCSD(T)-HOC. Up to this point, these represent our best set of results at the CC level with the ccECP and the DKH2 scalar relativistic Hamiltonian and the corresponding ccECP-pVXZ and cc-pVXZ-DK basis sets.

### Phaseless Auxiliary-Field Quantum Monte Carlo Results

Next, we tested phaseless auxiliary-field quantum Monte Carlo calculations. We employed several single- and multi-Slater determinant trial wave functions (SD and MSD), in accordance with best practice.^123−126^ This is a pragmatic and common approach to compute ph-AFQMC electronic energies because the best trial wave function is generally unknown (besides an FCI expansion, for which ph-AFQMC is exact). Since the solution for FeH is spin-contaminated to a large extent at the unrestricted Hartree–Fock level of theory,^23^ we decided to use restricted open-shell HF and KS methods to generate trial wave functions and alleviate any error propagated in ph-AFQMC due to spin-contamination.^127−130^ For the SH structure at the ROHF level of theory, the single-particle energies for the highest singly occupied molecular orbitals (HOMO) are always positive, regardless of the atomic basis set. However, the DFT HFLYP method stabilizes the HOMO of SH, which is our reason to include this approach in addition to HF. It is interesting to note that the HFLYP solutions resemble the ROHF total energies, i.e., the LYP correlation functional correlates electrons but gives molecular orbitals quite close to the corresponding canonical HF (100% exchange without LYP correlation) apart from HOMO (see Table S9 in the Supporting Information).

From a theoretical perspective, for a given basis set, the advantage of the MSD trial wave function is the convergence of the ph-AFQMC energy to a near-exact limit with an appropriately large increase in number of Slater determinants. Furthermore, the MSD wave function is the routine way to ascribe static correlation in a strongly correlated system. To generate an MSD trial wave functions with a predetermined number of Slater determinants, we first performed a heat-bath configuration interaction (HCI)^63,131^ calculation with an ε_1_ = 1 × 10^–4^ au threshold on top of the canonical ROHF wave functions. Subsequently, the resulting CI expansion was truncated to the desired number of determinants that contributed the most (based on the absolute value of the CI coefficient). The reference HCI energies are shown in Table S10 in the Supporting Information. In our work, due to rather high computational expenses, the maximum number of determinants was generally limited to 20 × 10^3^ and 15 × 10^3^ with double-ζ and mixed-ζ quality basis sets, respectively. A rather compact, orthogonal MSD trial wave function might lead to a large variation of the ph-AFQMC energy, but even a moderate number of determinants (∼10^4^) does not guarantee convergence of ph-AFQMC energies, neither to a plateau nor to the FCI limit.^62^ As a rule of thumb, it is always wise to go beyond single-determinant trial wave functions in the attempt to validate the reliability of the AFQMC energies.

As mentioned in the Computational Details Section, the all-electron ph-AFQMC calculations with Dunning’s DK basis sets were performed using the X2C Hamiltonian, while the CC calculations were performed using the DKH2 Hamiltonian. Although various scalar relativistic Hamiltonians result in different total electronic energies, the influence of the relativistic Hamiltonian on Δ*E* is negligibly small (less than 0.1 kJ/mol with HF and three CC methods; see Table S11 in the Supporting Information). Therefore, Δ*E* calculated with ph-AFQMC and CC can be compared without a loss of generality.

**Figure 5:**
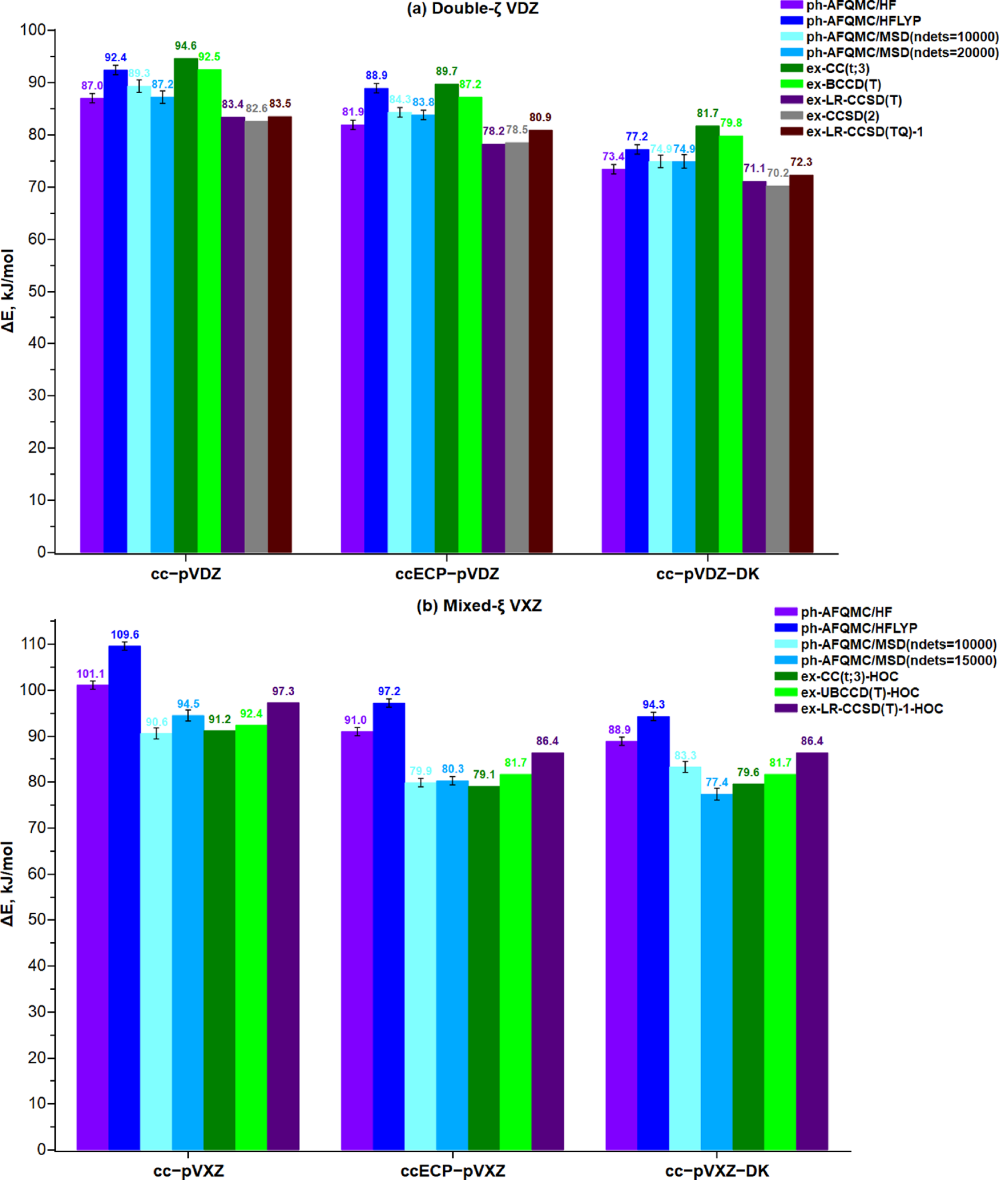
Nonrelativistic and scalar relativistic ph-AFQMC energy difference Δ*E* (kJ/mol) between the two model structures computed using different trial wave functions and (a) double-ζ quality or (b) mixed-ζ quality basis sets (in kJ/mol). The width of the error bars indicates the statistical uncertainties on the ph-AFQMC results.

To investigate the accuracy of the ph-AFQMC method, we first analyze the results obtained in nonrelativistic calculations with the cc-pVDZ basis set. As can be seen from Figure 5a (the raw data are shown in Table S12), the ph-AFQMC/cc-pVDZ Δ*E* results range from 87 ± 1 to 92 ± 1 kJ/mol with the SD HF and HFLYP trial wave functions giving the lowest and highest estimates, respectively, while the MSD results lie in between. More specifically, the ph-AFQMC approach with HF and MSD trial wave functions gives statistically compatible results between 87 ± 1 and 89 ± 1 kJ/mol. The SD HF and MSD ph-AFQMC/cc-pVDZ values agree fairly well with the extrapolated high-order ex-CCSD(2) and ex-LR-CCSD(TQ)-1 values, which are 83–84 kJ/mol. It is interesting to note that the corresponding extrapolated SHCI results are 87 ± 1 and 92 ± 3 kJ/mol with the linear and second-order polynomial fit, respectively.^23^ Moreover, the ph-AFQMC/MSD total electronic energies are in accordance with those of near-exact SHCI within chemical accuracy (Table S13 in the Supporting Information). Having such close agreement in the results between ph-AFQMC and near-exact deterministic high-level *ab initio* methods strengthens the confidence and applicability of ph-AFQMC for the given models and problem.

When relativistic effects are taken into account, the ph-AFQMC method also quantitatively reproduces the trend observed in CC, yielding a decrease in Δ*E* of 3–5 and 12–15 kJ/mol in the ECP and the all-electron calculation, respectively (with an uncertainty of 1–2 kcal/mol). Once again, as in the nonrelativistic case with the cc-pVDZ basis set, the SD and MSD trial ph-AFQMC results agree within chemical accuracy for both pseudopotential and all-electron calculations. Furthermore, increasing the CI expansion from 10000 to 20000 determinants yields statistically equivalent ph-AFQMC results (with differences of 0.5 ± 1 kJ/mol), which may be an indication that the MSD results are converged with respect to the size of the trial wave function. Compared to perturbative triples ex-CCq in double-ζ quality, the HFLYP trial wave function gives Δ*E* results closest to ex-UBCCD(T) (within 3 kJ/mol) whereas the MSD results are about 3–5 kJ/mol lower (with an uncertainty of 1 kJ/mol). Again, for double-ζ quality VDZ basis sets with or without scalar relativistic corrections, the 20000 MSD ph-AFQMC Δ*E* values are in excellent agreement with those of ex-LR-CCSD(TQ)-1. Specifically, the difference between MSD ph-AFQMC and extrapolated (TQ) CC results does not exceed 4 kJ/mol.

Due to the exponential increase in the dimension of the Hilbert space, the use of mixed-ζ quality VXZ basis sets can be somewhat more challenging as larger CI expansions might be required to converge the results with respect to the size of the trial wave function for the ph-AFQMC. Moreover, the failure of wave functions expanded in finite one-electron basis sets to reproduce electron–electron cusp effects poses additional complications. Consequently, we tested the use of trial wave functions with more than 15000 determinants with the double-ζ quality basis sets.

With the given computational constraints, the stochastic uncertainty in the energy difference Δ*E* increases slightly for the SD calculations (0.9–1.0 kJ/mol) and more significantly (up to 2.3 kJ/mol) for the MSD calculations with the VXZ basis set. As can be seen from Figure 5b, SD ph-AFQMC/HF provides Δ*E* values within 5 ± 1 kJ/mol of the ex-LR-CCSD(T)-HOC results in both the nonrelativistic and the relativistic case, while the ph-AQFMC/HFLYP results are 8–12 ± 1 kJ/mol higher than the ex-LR-CCSD(T)-HOC counterparts. The use of MSD trial wave functions clearly decreases Δ*E,* with the DK values for the expansion of 15000 determinant differing from the corresponding SD result by 12 ± 2 kJ/mol. We further validated the robustness of this estimate by increasing the expansion to 20000 and 25000 determinants, which resulted in an energy difference of 12–14 ± 2 kJ/mol with respect to ph-AQFMC/HF. Furthermore, we performed additional MSD ph-AFQMC/cc-pVXZ-DK calculations with the number of propagated blocks increased to 6500. In general, the MSD/ph-AFMQC Δ*E* remain almost the same and the most accurate ph-AFQMC result for Δ*E* with 25000 determinants is now 77.3 ± 1.9 kJ/mol. The differences in the total and relative energies for these two expansions are statistically indistinguishable (see Tables S14, S17, and S18 in the Supporting Information).

Consequently, the most accurate ph-AFQMC/MSD trial wave functions give Δ*E* results that are 4 and 9 kJ/mol lower than ex-UBCCD(T)-HOC and ex-LR-CCSD(T)-HOC, respectively. In other words, the most accurate ph-AFQMC/MSD trial wave functions and ex-UBCCD(T)-HOC give Δ*E* results that are in quantitative agreement within chemical accuracy. Such close agreement is quite interesting since only ex-UBCCD(T)-HOC and ex-CC(t;3)-HOC results include sizable high-order (TQ) CC energy corrections. Thus, the best estimates of Δ*E* at the ph-AFQMC level of theory are 80 ± 2 and 77 ± 2 kJ/mol with the ccECP-pVXZ and cc-pVXZ-DK basis sets, respectively. For these two Hamiltonians and the mixed-ζ quality basis sets, the small difference encountered between the ex-CCq-HOC (79–86 kJ/mol) and MSD ph-AFQMC (77–80 ± 2 kJ/mol) results might be due to the partial account of multiconfigurational effects either in the extrapolation of the CC results or in the trial wave function of ph-AFQMC. All HF and MSD ph-AFQMC results computed with the VXZ basis sets are listed in Tables S12–S18 in the Supporting Information.

### Basis-Set Incompleteness Error

Before comparing the wave function theory (WFT) *ab initio* results against DFT ones, we should estimate the magnitude of the basis-set incompleteness error (BSIE) for the former. Indeed, according to the literature, DFT results often converge rapidly with respect to the basis set contraction level, while WFT converges much slower. In other words, the WFT results might suffer from a considerable BSIE not present in the DFT calculations.^25^ To estimate BSIE, we performed a series of LR-CCSD(T) and UBCCD(T) calculations with the triple- to pentuple-ζ quality ccECP and Dunning’s DK basis sets. Table 5 lists the corresponding extrapolated CC Δ*E* energies along with the previously discussed mixed-ζ VXZ results.

**Table 5:** Energy Difference Δ*E* (kJ/mol) Computed from the Extrapolated CC Energies by Using Different Hamiltonians and Basis Sets^a^

		Δ*E* (kJ/mol)	
Hamiltonian	basis set	ex-UBCCD(T)	ex-LR-CCSD(T)
RECP	ccECP-pV*T*Z	85.3	79.3
RECP	ccECP-pVXZ	88.0	83.7
RECP	ccECP-pV*Q*Z	87.4	82.5
RECP	ccECP-pV*5*Z	90.6	86.4
X2C/DKH2	cc-pV*T*Z-DK	86.5	80.8
X2C/DKH2	cc-pVXZ-DK	89.2	85.4
X2C/DKH2	cc-pV*Q*Z-DK	88.1	83.7
X2C/DKH2	cc-pV*5*Z-DK	89.9	86.4

a The ex-UBCCD(T) and ex-LR-CCSD(T) results reported for the cc-pV{T/Q/5}Z-DK basis sets were computed using the X2C and DKH2 scalar relativistic Hamiltonian, respectively.

**Figure 6:**
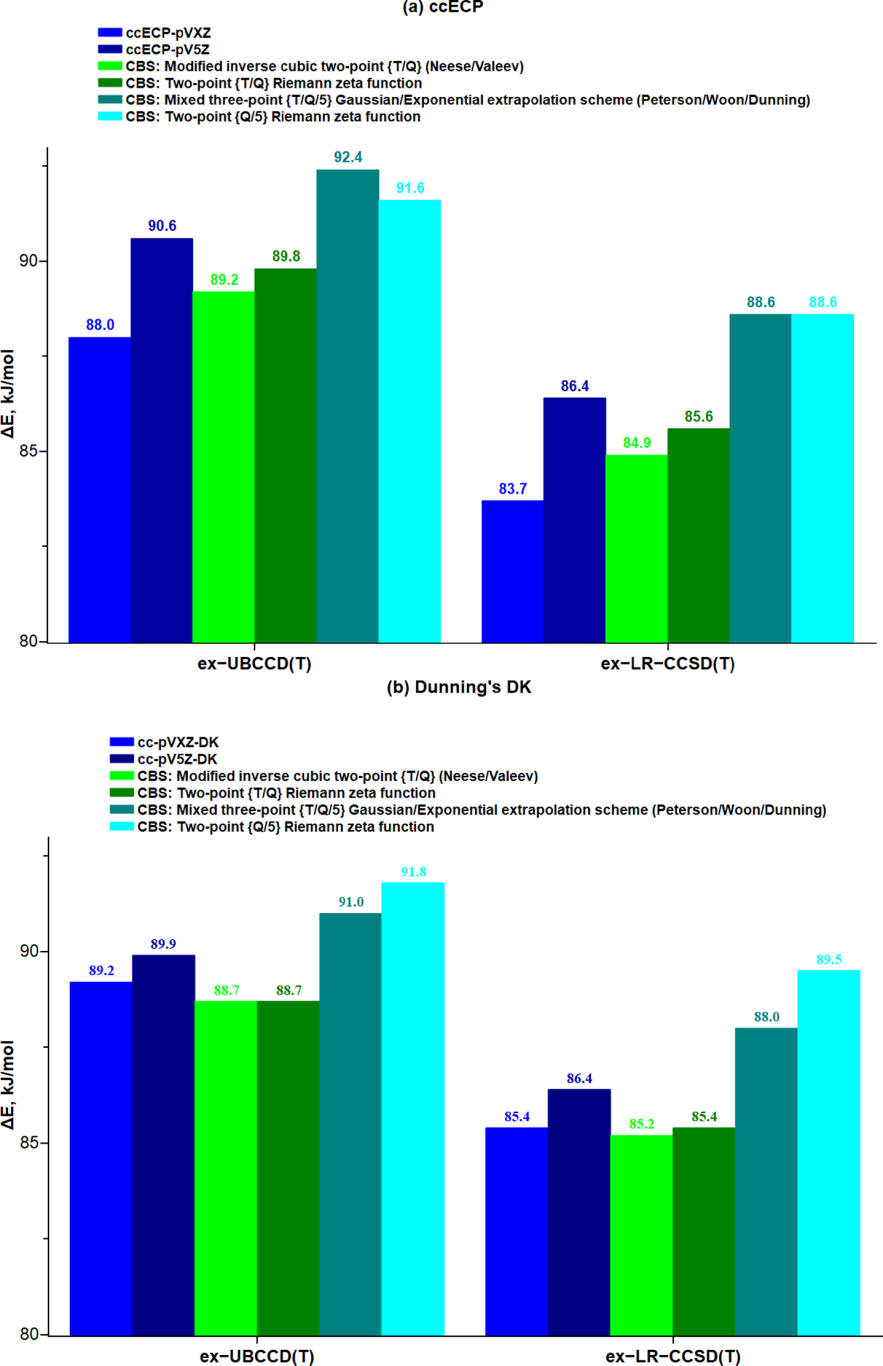
Scalar relativistic ex-UBCCD(T) and ex-LR-CCSD(T) energy difference Δ*E* (kJ/mol) extrapolated to the complete basis set limit (CBS). The ex-CCq results computed using the ccECP and the Dunning’s DK basis sets are visualized in the (a) top and (b) bottom panels, respectively. The Dunning’s DK UBCCD(T) and LR-CCSD(T) results were computed using X2C and DKH2 Hamiltonian, respectively.

Again, a systematic enlargement of the basis set within the mixed-ζ VXZ family causes a rather modest increase in Δ*E*. Specifically, by going from the cc-pVXZ-DK (ccECP-pVXZ) to the cc-pV5Z-DK (ccECP-pV*5*Z) basis set, ex-CC Δ*E* increases by 1 kJ/mol (3 kJ/mol) at most, although the number of basis functions increases from 274 (249) to 608 (583). Having the ex-CCq/cc-pV{T/Q/5}Z energies for each structure, one can perform a two- or three-point empirical extrapolation of the ex-CCq energies toward to the complete basis set limit (CBS). The final CBS ex-UBCCD(T) and ex-LR-CCSD(T) results are depicted in Figure 6 using four different extrapolation schemes. For each CC approach considered, the mixed-ζ VXZ ex-CCq results agree reasonably well with the corresponding CBS ones, within 5 kJ/mol both for the Dunning’s DK and the ccECP basis set family. We also observe that the extrapolation schemes using only the results with the triple- and quaduple-ζ basis set (green bars in Figure 6) give Δ*E* values that are ∼4 kJ/mol lower than those employing also the pentuple-ζ results. Furthermore, the CBS extrapolations up to pentuple-ζ give larger Δ*E* estimates than those obtained in the pure pV5Z calculations. Consequently, to account for the basis-set error, we simply increase our best ph-AFQMC/MSD and ex-CCq-HOC Δ*E* estimates by 3.6 kJ/mol, which is the average difference between the VXZ and the two CBS extrapolations employing the pentuple-ζ results. In addition, the all-electron Dunning’s DK Δ*E* results were increased by 1.3 kJ/mol to account for the CV correlation effects inherently covered by the all-electron DFT calculations. Our best reference WFT-based ex-CCq* and ph-AFQMC* estimates of Δ*E* are presented in Table 6.

**Table 6:** Reference Δ*E* Energy Differences Calculated with the MSD ph-AFQMC and CC Approaches and Two Scalar-Relativistic Basis Set Families (in kJ/mol)^a^

		reference Δ*E* (kJ/mol)		
Hamiltonian	basis set family	MSD ph-AFQMC*	ex-UBCCD(T)*	ex-LR-CCSD(T)*
RECP	ccECP	84 ± 2	85	90
DKH2/X2C	Dunning’s DK	82 ± 2	87	91

a For the all-electron Dunning’s DK basis set family, the ex-UBCCD(T)* and ex-LR-CCSD(T)* results were obtained by adding high-order (TQ), basis set incompleteness (BSIE), and core–valence (CV) energy corrections, whereas MSD ph-AFQMC* results were corrected by BSIE and CV only. For the ccECP basis set family, the ex-UBCCD(T)* and ex-LR-CCSD(T)* results were corrected by adding high-order (TQ) and BSIE corrections, whereas MSD ph-AFQMC* results were corrected by BSIE only.

We are now in a position to compare the reference results of Table 6 with DFT results computed using the scalar relavistic basis sets and Hamiltonians.

### DFT Results

To refine and revise the recommended list of DFT methods suitable for the two models, we calculated the Δ*E* energy difference with the mixed-ζ VXZ basis sets. The results with the nine DFT functionals are presented in Table 7, displaying a variation in Δ*E* from 68 to 133 kJ/mol (results with more functionals, including pure GGA and double hybrid functionals, were presented in our previous study;^23^ here we focused on the functionals that gave the best results).

**Table 7:** Δ*E* Energy Differences Calculated with Various Hybrid DFT Approaches and WFT Methods Using the Mixed-ζ Atomic Basis Sets (in kJ/mol)^a^

		Δ*E* (kJ/mol)		
		nonrelativistic	relativistic ECP	DKH2
method	%HF	cc-pVXZ	ccECP-pVXZ	cc-pVXZ-DK
DFT				
TPSSh	10	79.0	85.7	68.2
r^2^SCANh	10	86.5	75.6	75.0
O3LYP	12	92.6	93.7	81.8
B3LYP*	15	90.8	92.9	80.0
τ-HCTH	15	86.6	96.1	75.9
B3LYP	20	112.8	113.6	101.5
r^2^SCAN0	25	133.1	117.6	119.9
M06	27	98.3	92.3	87.6
M05	28	101.1	114.4	90.3
WFT				
MSD ph-AFQMC		94.5 ± 2.1	80.3 ± 2.3	77.2 ± 2.0
ex-BCCD(T)-HOC		92.4	81.7	81.7
ex-LR-CCSD(T)-HOC		97.3	86.4	86.4

a Note that no dispersion energy correction was included. %HF is the amount of the exact HF exchange contribution.

**Figure 7:**
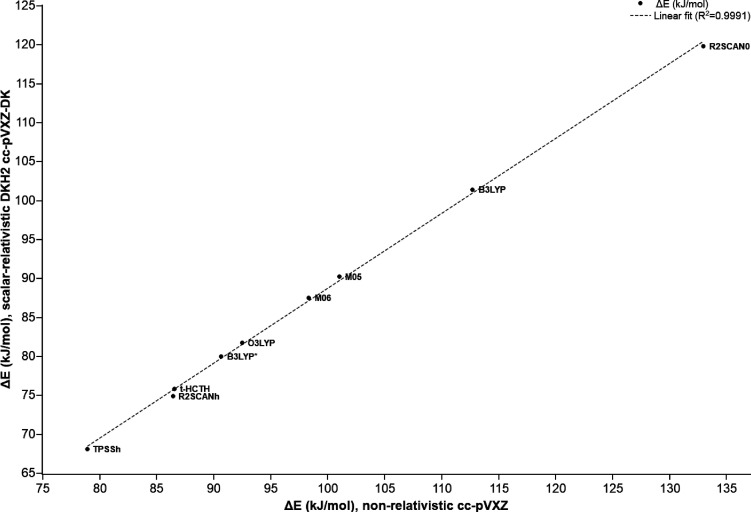
Energy difference Δ*E* (kJ/mol) between the two model structures calculated using nine hybrid DFT exchange–correlation functionals and two mixed-ζ all-electron atomic basis sets with (cc-pVXZ-DK) or without (cc-pVXZ) incorporating scalar relativistic effects.

**Figure 8:**
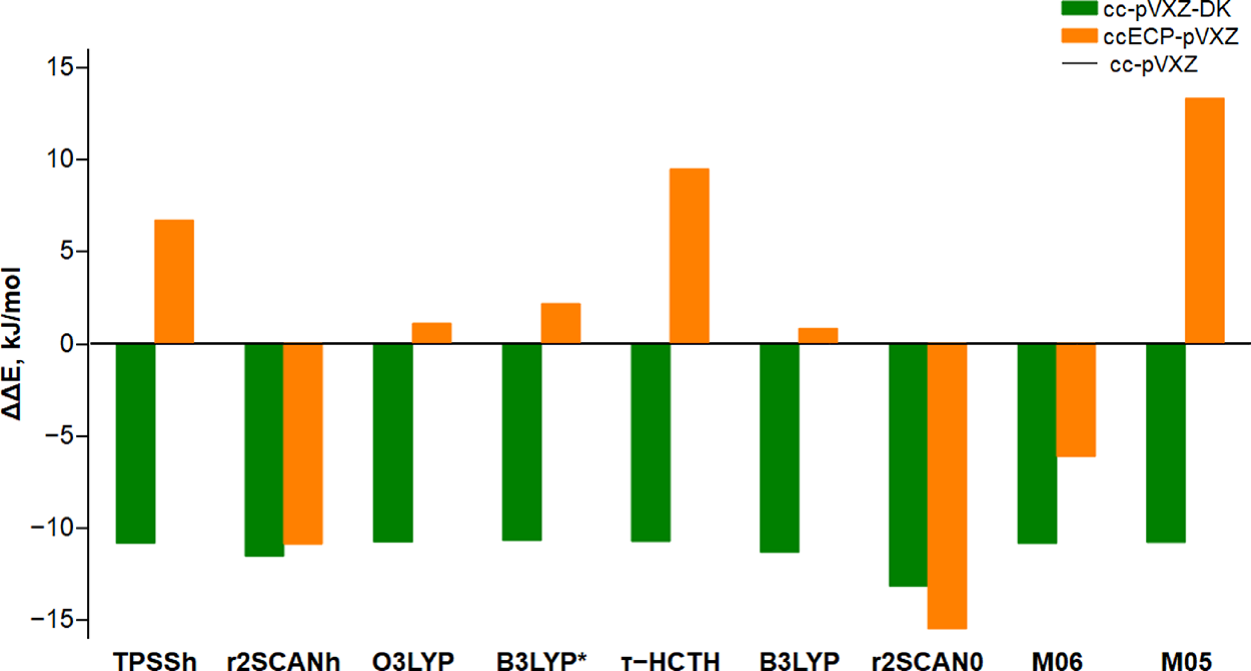
Energy changes in Δ*E* (kJ/mol) caused by scalar relativistic effects computed using nine DFT hybrid exchange–correlation functionals in all-electron (cc-pVXZ-DK) and pseudopotential (ccECP-pVXZ) calculations. The energy changes ΔΔ*E* are reported in kJ/mol relative to the corresponding DFT results obtained using the nonrelativistic all-electron cc-pVXZ basis set.

The DFT cc-pVXZ-DK Δ*E* results are consistently 11–13 kJ/mol lower than the corresponding non-relativistic values. In fact, there is a nearly perfect correlation between these two sets of results (*R*^2^ = 0.999) as is shown in Figure 7. In sharp contrast, the use of the ccECP pseudopotentials causes an ambiguous and non-systematic change of Δ*E* as is illustrated in Figure 8. In fact, Δ*E* increases by 7–13 kJ/mol for TPSSh, τ-HCTH, and M05, it remains roughly constant for O3LYP, B3LYP, and B3LYP*, and it decreases by 6–16 kJ/mol for M06, r^2^SCANh, and r^2^SCAN0. A possible explanation of the behavior of DFT/ccECP-pVXZ results is that the ccECP family of pseudopotentials and corresponding basis sets was originally developed and parametrized to reproduce many-body theories such as CCSD(T).^51,52^ It is intriguing that only the non-empirical family of r^2^SCAN based meta-GGA hybrids in combination with the ccECP pseudopotentials reproduces the all-electron relativistic DFT results. This is perhaps related to the fact that, among the functionals considered here, the original SCAN functional was the only one to be rigorously derived from first principles, obeying all 17 known exact constraints of meta-GGA.^87,90^ Indeed, it would be interesting to explore the practical implications of such finding for the real-space quantum Monte Carlo method, in particular, whether it is beneficial to use single-particle Kohn–Sham orbitals generated employing either r^2^SCAN-based hybrids or M06 when using ccECP pseudopotentials.

It can be seen from Table 7 that, for the all-electron cc-pVXZ-DK relativistic calculations, the M06 and M05 results (88 to 90 kJ/mol) are closest to our revised extrapolated ex-LR-CCSD(T)-HOC/cc-pVXZ-DK results while the O3LYP and B3LYP* (80 to 82 kJ/mol) approach to the ex-BCCD(T)-HOC ones. On the other hand, r^2^SCANh and τ-HCTH give Δ*E* results (75–76 kJ/mol) closest to our best MSD ph-AFQMC results with the cc-pVXZ-DK basis set, which include multiconfigurational effects. Hence, hybrid functionals with 10–15% HF exchange best mimic the high-level *ab initio* ph-AFQMC and CC results involving multiconfigurational effects or high-order (TQ) energy corrections. This finding is in agreement with previously reported results, based on geometries of Fe_2_ models.^17,18^ TPSSh and r^2^SCAN0 perform the worst and were excluded from further discussion.

Given the great success of the B3LYP* DFT method, with 15% exact HF exchange, in modeling transition-metal compounds and the robustness of r^2^SCAN, we decided to probe a combination of r^2^SCAN with 15% as well as 13% and 12% HF exchange. The new r^2^SCAN hybrids will be referred to as r^2^SCAN12, r^2^SCAN13, and r^2^SCAN15. We emphasize that a varying weight of HF exchange is not new but has been used by several scientists in conjunction within B3LYP^95,96,132−134^ and r^2^SCAN.^92^ However, to our best knowledge, we are the first to have combined r^2^SCAN with 12–15% HF exchange.

To perform a fair comparison of DFT with our best reference MSD ph-AFQMC* and ex-CCq* results, we performed two-point (two-parameter) CBS limit inverse cubic extrapolation of DFT energies obtained using quadruple- and pentuple-ζ quality Dunning’s DK basis sets and the DKH2 Hamiltonian. The a posteriori density-based VV10 dispersion energy corrections were included into standalone and thereby CBS DFT energies. Empirical D4 dispersion corrections were added directly to the DFT Δ*E* results extrapolated to the CBS limit. The DFT dispersion corrections were included for eight functionals for which parameters are available. The CBS DFT dispersion-corrected results are listed in Table 8.

**Table 8:** QZ/5Z CBS Δ*E* Energy Differences Calculated with the Various Hybrid DFT Exchange–Correlation (XC) Functionals with or without the D4 or VV10 Dispersion Corrections (in kJ/mol)

	Δ*E* (kJ/mol)		
XC	DFT	DFT-D4	DFT-VV10
r^2^SCANh	79.8	79.3	77.9
O3LYP	87.1	79.8	
B3LYP*	85.1	79.2^a^	75.5^a^
τ-HCTH	80.7		
r^2^SCAN12	86.0	85.5^a^	84.1^a^
r^2^SCAN13	89.1	88.6^a^	87.2^a^
r^2^SCAN15	95.2	94.7^a^	93.5^a^
B3LYP	106.3	100.4	96.4
M06	91.4	91.1	
M05	94.2		

a The damping parameters for B3LYP* and the new r^2^SCAN12/r^2^SCAN12/r^2^SCAN15 have not yet been defined, so we used the original damping parameters for B3LYP and r^2^SCANh.

It can be seen from Table 8 that the CBS extrapolation has a surprisingly large effect, increasing Δ*E* by 4–5 kJ/mol. Moreover, the D4 dispersion correction affects the M06 result and all r^2^SCAN-based results only slightly (<0.5 kJ/mol), while it lowers the O3LYP and B3LYP/B3LYP* results by 6–7 kJ/mol. Likewise, the density-based VV10 dispersion correction lowers all r^2^SCAN-based Δ*E* by 2 kJ/mol, but the B3LYP/B3LYP* results by 10 kJ/mol.

According to the results in Table 8, it appears that r^2^SCANh with and without dispersion, B3LYP*-D4, O3LYP-D4, and τ-HCTH give Δ*E* closest to the lower end of the reference MSD ph-AFQMC* results within 1 kJ/mol. Only r^2^SCAN12 with and without dispersion and B3LYP* approach the upper end of the MSD ph-AFQMC* reference within 2 kJ/mol. B3LYP*, O3LYP, and r^2^SCAN13 agree with the ex-UBCCD(T)* results within 2 kJ/mol. M06 with and without D4 correction gives a result that coincides with ex-LR-CCSD(T)*, whereas M05 gives a 3 kJ/mol larger Δ*E* estimate. The r^2^SCAN12 results Δ*E* range from 84 to 86 kJ/mol and thereby lie between the upper end of the MSD ph-AFQMC* and ex-BCCD(T)* references. To conclude, the r^2^SCAN with 10–13% HF exchange with and without dispersion, τ-HCTH, the O3LYP/O3LYP-D4, and the B3LYP*/B3LYP*-D4 methods generally perform the best among the DFT methods considered. In fact, the r^2^SCANh and r^2^SCAN13 quantitatively bracket the reference ph-AFQMC and CC estimates on Δ*E* to within 2 kJ/mol.

## Conclusions

We have studied the influence of scalar relativistic effects on the energy difference (Δ*E*) between two isomers of a minimal nitrogenase [Fe(SH)_4_H]^−^ model. By using CC and ph-AFQMC methods, we obtained several interesting results:

• We demonstrate that scalar relativistic effects play an important role and significantly reduce Δ*E* by ∼12 kJ/mol, primarily due to induced core–valence polarization.
• Canonical CCSD(T) with the HF reference exhibits the largest error (23–25 kJ/mol versus LR-CCSD(TQ)-1) among the tested CC methods with approximate triples, while advanced CC(t;3), UBCCD(T), UCC3, and LR-CCSD(T) approaches perform reasonably well.
• The BCCD(T) and CC(t;3) methods give nearly the same results for Δ*E* (within 1 kJ/mol), despite the vastly different underlying reference HF wave functions and formalisms.
• Post-perturbative triples high-order (TQ) CC energy corrections are important and decrease Δ*E* by 6–11 kJ/mol.
• The inner core–valence electron correlation effects increase Δ*E* by only 1 kJ/mol.
• The mixed-ζ quality VXZ basis sets are accurate and provide results that are only 2 and 4 kJ/mol lower compared to pentuple-ζ quality basis set and CBS extrapolations, respectively.
• With coupled cluster calculations extrapolated toward FCI, the best estimates for Δ*E*, including relativistic, high-order (TQ) CC, core–valence correlation, and basis set incompleteness corrections are 87–91 kJ/mol and 85–90 with all-electron and ECP basis sets, respectively.
• The stochastic MSD ph-AFQMC method predicts Δ*E* in the range from 82 ± 2 to 84 ± 2 kJ/mol with scalar relativistic all-electron and ECP basis sets, respectively.
• The reference scalar relativistic MSD ph-AFQMC* estimates differ from the corresponding ex-BCCD(T)* values by 5 ± 2 and 1 ± 2 kJ/mol with the all-electron and ECP basis sets, respectively. This could be due to multiconfigurational effects.
• Single-determinant ph-AFQMC calculations with an ROHF trial wave function provides Δ*E* values within 5 ± 1 kJ/mol of the corresponding extrapolated ex-LR-CCSD(T)-HOC coupled-cluster results. The latter approach is in practice missing the high-order (TQ) energy corrections.
• We introduced a new meta-GGA hybrid, r^2^SCAN with 12% HF exchange called r^2^SCAN12. It reproduces the reference MSD ph-AFQMC* and ex-BCCD(T)* Δ*E* results within 2 kJ/mol.
• r^2^SCAN with 10–13% HF exchange (with and without dispersion), τ-HCTH, O3LYP/O3LYP-D4, and B3LYP*/B3LYP*-D4 methods generally perform the best among the DFT methods considered.
• The DFT results are sensitive to the choice of the exchange–correlation functional and dispersion corrections and to the use of ECPs. Increasing the basis set from the mixed-ζ quality VXZ to a {*Q/*5} CBS extrapolation increases the DFT Δ*E* energies by 4–5 kJ/mol.

## Supporting Information Available

Complete basis set specification; HF and CC energies employing different Hamiltonians and basis sets; energy difference Δ*E* computed on top of extrapolated CC energies; reference trial single-determinant (HF and HFLYP) wave function energies; reference SHCI energies computed with ε_1_ = 1 × 10^–4^ threshold; unrestricted CC energies computed employing the cc-pVXZ-DK basis set with the DKH2 and X2C scalar relativistic Hamiltonians; energy difference Δ*E* computed using ph-AFQMC with different trial wave functions and basis sets (PDF)
