# Supplementary material for: Scalar Relativistic All-Electron and Pseudopotential Ab Initio Study of a Minimal Nitrogenase [Fe(SH)4H]− Model Employing Coupled-Cluster and Auxiliary-Field Quantum Monte Carlo Many-Body Methods
Source: J Phys Chem A. 2024 Feb 7;128(7):1358–74. doi: 10.1021/acs.jpca.3c05808 (PMC10895656; doi:10.1021/acs.jpca.3c05808)
Supplement: Supplementary file 1 — jp3c05808_si_001.pdf [file jp3c05808_si_001.pdf]

Supporting information

Scalar Relativistic All-electron and Pseudopotential *ab initio* Study of a Minimal Nitrogenase $[Fe(SH)_4H]^-$ Model Employing Coupled-cluster and Auxiliary-field Quantum Monte Carlo Many-body Methods

Victor P. Vysotskiy[a], Claudia Filippi[b] and Ulf Ryde[a]**

[a] Department of Computational Chemistry, Lund University, Chemical Centre, SE-221 00 Lund, Sweden

[b] MESA+ Institute for Nanotechnology, University of Twente, P.O. Box 217, 7500 AE Enschede, Netherlands

2023-12-04

Table S1. Specification of the basis sets used in the present study. The (-DK) basis sets were used together with either the DKH2 or the X2C scalar relativistic Hamiltonian.

Basis set	Number of basis functions	Number of frozen-core electrons (post-HF)	Atomic center			
			H (terminating)	H^+ (reactive)	Fe	S
ccECP-pVDZ	115	0	ccECP-pVDZ	ccECP-pVDZ	ccECP-pVDZ	ccECP-pVDZ
ccECP-pVXZ	249	0	ccECP-pVDZ	ccECP-pVTZ	ccECP-pVQZ	ccECP-pVTZ
ccECP-pV*T*Z	213	0	ccECP-pVDZ	ccECP-pVTZ	ccECP-pVTZ	ccECP-pVTZ
ccECP-pV*Q*Z	365	0	ccECP-pVDZ	ccECP-pVQZ	ccECP-pVQZ	ccECP-pVQZ
ccECP-pV*5*Z	583	0	ccECP-pVDZ	ccECP-pV5Z	ccECP-pV5Z	ccECP-pV5Z
cc-pVDZ(-DK)	140	50	cc-pVDZ(-DK)	cc-pVDZ(-DK)	cc-pVDZ(-DK)	cc-pVDZ(-DK)
cc-pVXZ(-DK)	274	50	cc-pVDZ(-DK)	cc-pVDZ(-DK)	cc-pVQZ(-DK)	cc-pVTZ(-DK)
cc-pCVXZ-DK	419	10 or 50	cc-pVDZ(-DK)	cc-pVDZ(-DK)	cc-pwCVQZ-DK	cc-pCVTZ-DK
cc-pV*T*Z-DK	238	50	cc-pVDZ-DK	cc-pVTZ-DK	cc-pVTZ-DK	cc-pVTZ-DK
cc-pV*Q*Z-DK	390	50	cc-pVDZ-DK	cc-pVQZ-DK	cc-pVQZ-DK	cc-pVQZ-DK
cc-pV*5*Z-DK	608	50	cc-pVDZ-DK	cc-pV5Z-DK	cc-pV5Z-DK	cc-pV5Z-DK

Table S2. Non-relativisitc HF and CC energies (a.u.) employing Dunning's basis sets. The UCCSD(T) results are taken from Ref. 1.

Basis set	cc-pVDZ		cc-pVXZ	
	FeH	SH	FeH	SH
ROHF	-2855.515295	-2855.668025	-2855.580543	-2855.732009
ROCCSD	-2856.636305	-2856.695097	-2857.117162	-2857.184243
CR-CC(2,3)	-2856.685849	-2856.726088	-2857.197009	-2857.241831
CC(t;3)	-2856.687346	-2856.726560	-2857.198685	-2857.242467
UHF	-2855.569665	-2855.675487	-2855.638332	-2855.740674
UCCSD	-2856.636532	-2856.694779	-2857.118383	-2857.184510
UBCCD(T)	-2856.687449	-2856.726121	-2857.201279	-2857.245036
LR-CCSD(T)	-2856.690398	-2856.726143	-2857.202362	-2857.244812
CCSD(2)	-2856.691892	-2856.727519		
LR-CCSD(TQ)-1	-2856.693274	-2856.725399		
UCCSD(T)[1]	-2856.683320	-2856.724958		

Table S3. HF and CC energies (a.u.) employing ccECP pseudopotentials and corresponding basis sets. Note that the restricted open-shell CR-CC(2.3) and CC(t;3) results were computed without the *h*-type basis functions for the ccECP-pVXZ basis set, i.e., the original ccECP-pVXZ basis set was truncated to the *spdfg* subset. However, the LR-CCSD(T) results were computed with the non-truncated original ccECP-pVXZ basis set. Therefore, two sets of ROCCSD/ccECP-pVXZ energies are reported, viz. without and with *h*-type functions, marked with * and **, respectively.

Basis set	ccECP-pVDZ		ccECP-pVXZ	
	FeH	SH	FeH	SH
ROHF	-165.408746	-165.557137	-165.451850	-165.597461
ROCCSD	-166.555780	-166.612492	-167.029871*<br>-167.045627**	-167.091068*<br>-167.107508**
CR-CC(2,3)	-166.603774	-166.641870	-167.111543	-167.150737
CC(t;3)	-166.605176	-166.642297	-167.113147	-167.151363
UHF	-165.464863	-165.565542	-165.512383	-165.607205
UCCSD	-166.556517	-166.612503	-167.046567	-167.107523
UBCCD(T)	-166.605681	-166.642095	-167.132359	-167.171031
LR-CCSD(T)	-166.608853	-166.642377	-167.134065	-167.171329
CCSD(2)	-166.609878	-166.643600		
LR-CCSD(TQ)-1	-166.610784	-166.641729		
UCCSD(T)	-166.601419	-166.641022		

Table S4. HF and CC energies (a.u.) employing the second-order Douglas–Kroll Hamiltonian and the corresponding scalar relativistic Dunning's basis sets. Note that the restricted open-shell CR-CC(2.3) and CC(t;3) results were computed without the *h*-type basis functions for the cc-pVXZ-DK basis set, i.e., the original cc-pVXZ-DK basis set was truncated to the *spdfg* subset. However, the LR-CCSD(T) results were computed with the non-truncated original cc-pVXZ-DK basis set. Therefore, two sets of ROCCSD/cc-pVXZ-DK energies are reported, viz. without and with *h*-type functions, marked with * and **, respectively.

Basis set	cc-pVDZ-DK		cc-pVXZ-DK	
	FeH	SH	FeH	SH
ROHF	-2868.724145	-2868.869670	-2868.793650	-2868.937839
ROCCSD	-2869.846436	-2869.899725	-2870.315580* -2870.329946**	-2870.376508* -2870.391546**
CR-CC(2,3)	-2869.895696	-2869.930887	-2870.393999	-2870.433471
CC(t;3)	-2869.897153	-2869.931356	-2870.395626	-2870.434097
UHF	-2868.779871	-2868.877141	-2868.852653	-2868.946478
UCCSD	-2869.846774	-2869.899413	-2870.331451	-2870.391995
UBCCD(T)	-2869.897275	-2869.930987	-2870.413896	-2870.452849
LR-CCSD(T)	-2869.900031	-2869.930954	-2870.414921	-2870.452648
CCSD(2)	-2869.901604	-2869.932357		
LR-CCSD(TQ)-1	-2869.902439	-2869.930163		
UCCSD(T)	-2869.893093	-2869.929772		
UCC3	-2869.904526	-2869.933566		

Table S5. The energy difference ΔE (kJ/mol) computed with post-(T) CCSD(2) and LR-CCSD(TQ)-1 approaches using the various Hamiltonians and double-ζ basis sets. The canonical CC and extrapolated results are given in the first two and last two columns (prefixed via -ex), respectively.

Basis set	ΔE (kJ/mol)			
	CCSD(2)	LR-CCSD(TQ)-1	ex-CCSD(2)	ex-LR-CCSD(TQ)-1
cc-pVDZ	93.5	84.3	82.8	83.5
ccECP-pVDZ	88.5	81.2	78.5	80.9
cc-pVDZ-DK	80.7	72.8	70.4	72.3

Table S6. All-electron CC energy difference ΔE computed using the scalar relativistic DKH2 Hamiltonian and polarized core-valence cc-pCVXZ-DK basis set with either 50 or 10 frozen core electrons. For each CC method reported, the energy correlation effect (CV) between subvalence core – semi-core plus valence electrons is estimated as net difference in CC ΔE computed keeping frozen 50 and 10 electrons. The resulting CV energy corrections are 1.3 and 1.2 kJ/mol for the UBCCD(T), and LR-CCSD(T) methods, respectively.

Structure	Total energy (a.u.)			
	50 electrons frozen		10 electrons frozen	
	UBCCD(T)	LR-CCSD(T)	UBCCD(T)	LR-CCSD(T)
FeH	-2870.490617	-2870.478264	-2871.936866	-2871.924959
SH	-2870.530841	-2870.517633	-2871.977591	-2871.964795
ΔE (kJ/mol)	105.6	103.4	106.9	104.6

Table S7. The energy difference ΔE (kJ/mol) computed from the extrapolated CC energies using the various Hamiltonians and basis sets.

Basis set	ΔE (kJ/mol)		
	ex-CC(t;3)	ex-UBCCD(T)	ex-LR-CCSD(T)
	Double-ζ basis sets		
cc-pVDZ	94.6	92.5	83.6
ccECP-pVDZ	89.7	87.2	78.2
cc-pVDZ-DK	81.7	79.8	71.3
	Mixed-ζ basis sets		
cc-pVXZ	102.3	101.4	97.4
ccECP-pVXZ	87.9	88.0	83.7
cc-pVXZ-DK	89.0	89.2	85.4

Table S8. The a posteriori HOC corrected ΔE (kJ/mol) computed from the extrapolated CC energies using the various Hamiltonians and mixed-ζ VXZ basis sets.

Basis set	ΔE (kJ/mol)		
	ex-CC(t;3)-HOC	ex-UBCCD(T)-HOC	ex-LR-CCSD(T)-HOC
cc-pVXZ	91.2	92.4	97.3
ccECP-pVXZ	79.1	81.7	86.4
cc-pVXZ-DK	79.6	81.7	86.4

Table S9. Total energies and energy differences computed at the HF and DFT HFLYP levels with various basis sets. The results denoted "KS HFLYP orbitals" correspond to the non-self-consistent ROHF energies computed using molecular orbitals results from solving the ROKS DFT equations self-consistently. The results denoted "HF orbitals" and "DFT HFLYP" correspond to fully self-consistent calculations. Note that the results reported for both the cc-pVDZ-DK and cc-pVXZ-DK basis sets were computed using the X2C scalar relativistic Hamiltonian.

Basis set	Energy	HF orbitals	KS HFLYP orbitals	DFT HFLYP
cc-pVDZ	FeH, a.u.	-2855.515292	-2855.51398	-2859.490791
	SH, a.u.	-2855.668022	-2855.66679	-2859.638802
	ΔE, kJ/mol	401.0	401.2	388.6
ccECP-pVDZ	FeH, a.u.	-165.408743	-165.407859	-166.797857
	SH, a.u.	-165.557135	-165.556375	-166.940712
	ΔE, kJ/mol	389.6	389.9	375.1
cc-pVDZ-DK	FeH, a.u.	-2868.735552	-2868.734241	-2872.711724
	SH, a.u.	-2868.881074	-2868.879841	-2872.852643
	ΔE, kJ/mol	382.1	382.3	370.0
cc-pVXZ	FeH, a.u.	-2855.580542	-2855.579083	-2859.555302
	SH, a.u.	-2855.732008	-2855.73063	-2859.702153
	ΔE, kJ/mol	397.7	397.9	385.6
ccECP-pVXZ	FeH, a.u.	-165.451853	-165.450939	-166.840954
	SH, a.u.	-165.597466	-165.596664	-166.981125
	ΔE, kJ/mol	382.3	382.6	368.0
cc-pVXZ-DK	FeH, a.u.	-2868.805691	-2868.804234	-2872.781133
	SH, a.u.	-2868.949877	-2868.948500	-2872.920832
	ΔE, kJ/mol	378.6	378.8	366.8

Table S10. Reference heat bath configuration interaction energies (a.u.) computed employing different basis sets and $\varepsilon_1 = 1 \times 10^{-4}$. The resulting number of determinants (ndets) is also reported for each structure and basis set.

Basis set	FeH		SH	
	Total energy (a.u.)	ndets	Total energy (a.u.)	ndets
cc-pVDZ	-2856.391350	7 156 226	-2856.476915	5 647 455
ccECP-pVDZ	-166.316281	7 823 582	-166.395785	5 801 750
cc-pVDZ-DK	-2869.610635	6 291 031	-2869.689997	4 979 561
cc-pVXZ	-2856.732500	11 213 325	-2856.837670	14 107 738
ccECP-pVXZ	-166.648351	11 505 131	-166.744954	14 127 482
cc-pVXZ-DK	-2869.957691	11 024 221	-2870.056413	14 228 830

Table S11. Unrestricted CC energies (a.u) and energy differences (kJ/mol) computed employing the cc-pVXZ-DK basis set with the DKH2 and X2C scalar relativistic Hamiltonians.

Method	Scalar relativistic Hamiltonian	Electronic energy (a.u.)		ΔE (kJ/mol)
		FeH	SH	
UHF	DKH2	-2868.852653	-2868.946478	246.3
	X2C	-2868.864692	-2868.958514	246.3
UCCSD	DKH2	-2870.331451	-2870.391995	159.0
	X2C	-2870.343486	-2870.404028	159.0
UBCCD(T)	DKH2	-2870.413896	-2870.452849	102.3
	X2C	-2870.425930	-2870.464883	102.3
ex-UBCCD(T)	DKH2	-2870.424610	-2870.458585	89.2
	X2C	-2870.436645	-2870.470619	89.2

Table S12. Energy difference ΔE (kJ/mol) computed using ph-AFQMC with different Hamiltonians and trial wavefunctions, and the double-ζ quality basis sets. ndets is the number of Slater determinants.

Trial wavefunction	Basis set		
	cc-pVDZ	ccECP-pVDZ	cc-pVDZ-DK
HF	87.0 ± 0.9	81.9 ± 0.9	73.4 ± 0.9
HFLYP	92.4 ± 0.9	88.9 ± 0.9	77.2 ± 0.9
MSD (ndets=10000)	89.3 ± 1.2	84.3 ± 0.9	74.9 ± 1.2
MSD (ndets=20000)	87.2 ± 1.2	83.8 ± 0.9	74.9 ± 1.3

Table S13. Non-relativistic ph-AFQMC and the SHCI total electronic energies (a.u.) extrapolated to the FCI limit computed using the cc-pVDZ basis set. The SHCI results are taken from Ref. [1]

Method	E_{Fe-H}	E_{S-H}
SHCI (1st order polynominal fitting)[1]	-2856.696603 ± 0.000174	-2856.729634 ± 0.000207
SHCI (2nd order polynominal fitting)[1]	-2856.697439 ± 0.001172	-2856.732414 ± 0.000253
ph-AFQMC/MSD (ndets=10000)	-2856.698014 ± 0.000361	-2856.732035 ± 0.000301
ph-AFQMC/MSD (ndets=20000)	-2856.698891 ± 0.000377	-2856.732117 ± 0.000226

Table S14. Energy difference ΔE (kJ/mol) computed using ph-AFQMC with different Hamiltonians and trial wavefunctions, and the mixed-ζ quality basis sets. ndets is the number of Slater determinants.

Trial wavefunction	Basis set		
	cc-pVXZ	ccECP-pVXZ	cc-pVXZ-DK
HF	101.1 ± 0.9	91.0 ± 1.0	88.9 ± 1.0
HFLYP	109.6 ± 1.0	97.2 ± 1.0	94.3 ± 1.0
MSD (ndets=10000)	90.6 ± 2.3	79.9 ± 1.8	83.3 ± 2.2
MSD (ndets=15000)	94.5 ± 2.1	80.3 ± 2.3	77.4 ± 1.8
MSD (ndets=20000)	–	–	75.0 ± 1.8
MSD (ndets=25000)	–	–	77.2 ± 2.0

Table S15. Non-relativistic ph-AFQMC total electronic energies (a.u.) computed using cc-pVXZ basis set.

Trial wavefunction	E_{Fe-H}	E_{S-H}
ph-AFQMC/ROHF	-2857.215277 ± 0.000280	-2857.253797 ± 0.000228
ph-AFQMC/MSD (ndets=10000)	-2857.217122 ± 0.000714	-2857.251647 ± 0.000516
ph-AFQMC/MSD (ndets=15000)	-2857.217293 ± 0.000716	-2857.253270 ± 0.000349

Table S16. The ph-AFQMC total electronic energies (a.u.) computed using the ccECP pseudopotentials and corresponding ccECP-pVXZ basis set. The spin-projected[2] ph-AFQMC/UHF total electronic energies (a.u.) computed using an original approach by Zhang et al.[2]

Trial wavefunction	E_{Fe-H}	E_{S-H}
ph-AFQMC/sp-UHF	-166.648720 ± 0.000214	-167.179762 ± 0.000242
ph-AFQMC/ROHF	-167.144514 ± 0.000302	-167.179161 ± 0.000240
ph-AFQMC/MSD (ndets=10000)	-167.147019 ± 0.000550	-167.177468 ± 0.000408
ph-AFQMC/MSD (ndets=15000)	-167.146998 ± 0.000768	-167.177570 ± 0.000409

Table S17. The ph-AFQMC total electronic energies (a.u.) computed using cc-pVXZ-DK basis set and scalar relativistic X2C Hamiltonian.

Trial wavefunction	$E_{Fe\text{-}H}$	$E_{S\text{-}H}$
ph-AFQMC/ROHF	-2870.438994 ± 0.000293	-2870.472838 ± 0.000224
ph-AFQMC/MSD (ndets=10000)	-2870.440593 ± 0.000639	-2870.472311 ± 0.000535
ph-AFQMC/MSD (ndets=15000)	-2870.441823 ± 0.000568	-2870.471299 ± 0.000353
ph-AFQMC/MSD (ndets=20000)	-2870.442053 ± 0.000559	-2870.470601 ± 0.000406
ph-AFQMC/MSD (ndets=25000)	-2870.441550 ± 0.000659	-2870.470939 ± 0.000347

Table S18. Total ph-AFMQC electronic energies and ΔE energy differences (kJ/mol) computed with the X2C Hamiltonian, the MSD trial wavefunctions, and the mixed-ζ quality cc-pVXZ-DK basis set. The number of propagated blocks was set to 6500.

Trial wavefunction	Electronic energy (a.u.)		ΔE
	$E_{Fe\text{-}H}$	$E_{S\text{-}H}$	(kJ/mol)
MSD (ndets=20000)	-2870.441657 ± 0.000421	-2870.470453 ± 0.000306	75.6 ± 1.4
MSD (ndets=25000)	-2870.441544 ± 0.000643	-2870.470995 ± 0.000316	77.3 ± 1.9

Table S19. Scalar-relativisitc UHF, UCCSD, and BCCD(T) energies (a.u.) employing Dunning's DK and ccECP basis sets. Note that the results reported for the cc-pV*Y*Z-DK basis sets (*Y* = *T*, *Q*, or *5*) were computed using the X2C scalar relativistic Hamiltonian.

Basis set	UHF		UCCSD		UBCCD(T)	
	FeH	SH	FeH	SH	FeH	SH
ccECP-pV*T*Z	-165.509638	-165.605003	-166.970533	-167.029904	-167.051331	-167.088796
ccECP-pV*Q*Z	-165.519916	-165.615017	-167.123362	-167.184672	-167.217631	-167.256530
ccECP-pV*5*Z	-165.524217	-165.620323	-167.188592	-167.251663	-167.288507	-167.328865
cc-pV*T*Z-DK	-2868.860572	-2868.954488	-2870.262183	-2870.321080	-2870.340024	-2870.377798
cc-pV*Q*Z-DK	-2868.883683	-2868.978103	-2870.425056	-2870.485686	-2870.514943	-2870.553851
cc-pV*5*Z-DK	-2868.890993	-2868.985535	-2870.497429	-2870.559161	-2870.592502	-2870.632290

Table S20. Scalar-relativisitc ROHF, ROCCSD, and LR-CCSD(T) energies (a.u.) employing Dunning's DK and ccECP basis sets. Note that the results reported for the cc-pV*Y*Z-DK basis sets (*Y* = *T*, *Q*, or *5*) were computed using the DKH2 scalar relativistic Hamiltonian.

Basis set	ROHF		ROCCSD		LR-CCSD(T)	
	FeH	SH	FeH	SH	FeH	SH
ccECP-pV*T*Z	-165.449474	-165.595495	-166.969574	-167.029890	-167.053508	-167.089040
ccECP-pV*Q*Z	-165.459323	-165.605253	-167.122326	-167.184657	-167.219438	-167.256752
ccECP-pV*5*Z	-165.463679	-165.610484	-167.187574	-167.251650	-167.290138	-167.329108
cc-pV*T*Z-DK	-2868.790346	-2868.934559	-2870.249162	-2870.309125	-2870.330048	-2870.366013
cc-pV*Q*Z-DK	-2868.812432	-2868.957241	-2870.411275	-2870.473051	-2870.503903	-2870.541420
cc-pV*5*Z-DK	-2868.819153	-2868.964073	-2870.483146	-2870.546028	-2870.580721	-2870.619388

Table S21. The extrapolated energies to the complete basis set limit (CBS) for ex-UBCCD(T) and ex-LR-CCSD(T) employing Dunning's DK sets. Note that the ex-BCCD(T) and ex-LR-CCSD(T) results were computed using the X2C and DKH2 scalar relativistic Hamiltonian, respectively. In the case of Riemann zeta function extrapolation scheme, a unified-single-parameter-extrapolation scheme by Varandas et al. was used to extrapolate Hartree-Fock energies individually.[3]

CBS extrapolation scheme	Number of points	ex-UBCCD(T)		ex-LR-CCSD(T)	
		FeH	SH	FeH	SH
Modified inverse cubic (Neese/Valeev)[4]	Two – {*T/Q*}Z	-2870.643889	-2870.677659	-2870.632722	-2870.665154
Riemann zeta function[5]	Two – {*T/Q*}Z	-2870.674963	-2870.708749	-2870.663773	-2870.696306
A Mixed Gaussian/Exponential Extrapolation Scheme (Peterson/Woon/Dunning)[6]	Three – {*T/Q/5*}Z	-2870.651585	-2870.686228	-2870.639527	-2870.673059
Riemann zeta function[5]	Two – {*Q/5*}Z	-2870.694686	-2870.729639	-2870.682729	-2870.716807

Table S22. The extrapolated energies to the complete basis set limit (CBS) for ex-UBCCD(T) and ex-LR-CCSD(T) employing ccECP basis sets. In the case of Riemann zeta function extrapolation scheme, a unified-single-parameter-extrapolation scheme by Varandas et al. was used to extrapolate Hartree-Fock energies individually.[3]

CBS extrapolation scheme	Number of points	ex-UBCCD(T)		ex-LR-CCSD(T)	
		FeH	SH	FeH	SH
Modified inverse cubic (Neese/Valeev)[4]	Two – {*T/Q*}Z	-167.346716	-167.3806991	-167.348653	-167.381004
Riemann zeta function[5]	Two – {*T/Q*}Z	-167.378778	-167.4129782	-167.380710	-167.413330
A Mixed Gaussian/Exponential Extrapolation Scheme (Peterson/Woon/Dunning)[6]	Three – {*T/Q/5*}Z	-167.344684	-167.3798856	-167.346396	-167.380145
Riemann zeta function[5]	Two – {*Q/5*}Z	-167.386805	-167.421701	-167.388301	-167.422055

Table S23. Bond lengths to Fe (in Å) of the $[Fe(SCH_3)_4H]^-$ model obtained with different DFT methods, basis sets, and with or without a relativistic Hamiltonian (Rel). The last two columns show ΔE in kJ/mol, calculated either with the same method as the optimization (ΔE_1) or with the same method for all entries (non-relativistic TPSS or B3LYP and the def2-TZVPD basis set; ΔE_2).

DFT	Basis	Rel	FeH structure					SH structure				ΔE_1	ΔE_2
			S1	S2	S3	S4	H	S1	S2	S3	SH_2		
TPSS	def2-SV(P)	no	2.27	2.31	2.31	2.26	1.56	2.29	2.32	2.32	2.49	-9.4	16.5
B3LYP			2.30	2.33	2.33	2.30	1.56	2.33	2.35	2.36	2.64	68.6	89.8
TPSS	def2-TZVPD	no	2.25	2.30	2.29	2.25	1.54	2.27	2.29	2.29	2.42	19.6	19.6
B3LYP			2.29	2.32	2.32	2.29	1.53	2.32	2.33	2.34	2.60	91.7	91.7
TPSS	x2c-TZVPAll	X2C	2.24	2.29	2.29	2.24	1.54	2.26	2.28	2.28	2.41	8.4	19.6
B3LYP			2.28	2.31	2.31	2.28	1.53	2.31	2.32	2.33	2.59	77.0	91.5

References

(1) Vysotskiy, V. P.; Torbjornsson, M.; Jiang, H.; Larsson, E. D.; Cao, L.; Ryde, U.; Zhai, H.; Lee, S.; Chan, G. K. Assessment of DFT functionals for a minimal nitrogenase [Fe(SH)4H]- model employing state-of-the-art ab initio methods. *J Chem Phys* **2023**, *159* (4). DOI: 10.1063/5.0152611 From NLM Publisher.

(2) Purwanto, W.; Al-Saidi, W. A.; Krakauer, H.; Zhang, S. Eliminating spin contamination in auxiliary-field quantum Monte Carlo: realistic potential energy curve of F(2). *J Chem Phys* **2008**, *128* (11), 114309. DOI: 10.1063/1.2838983 From NLM PubMed-not-MEDLINE.

(3) Pansini, F. N. N.; Neto, A. C.; Varandas, A. J. C. Extrapolation of Hartree–Fock and multiconfiguration self-consistent-field energies to the complete basis set limit. *Theoretical Chemistry Accounts* **2016**, *135* (12), 261. DOI: 10.1007/s00214-016-2016-4.

(4) Neese, F.; Valeev, E. F. Revisiting the Atomic Natural Orbital Approach for Basis Sets: Robust Systematic Basis Sets for Explicitly Correlated and Conventional Correlated ab initio Methods? *J Chem Theory Comput* **2011**, *7* (1), 33-43. DOI: 10.1021/ct100396y From NLM PubMed-not-MEDLINE.

(5) Lesiuk, M.; Jeziorski, B. Complete Basis Set Extrapolation of Electronic Correlation Energies Using the Riemann Zeta Function. *Journal of Chemical Theory and Computation* **2019**, *15* (10), 5398-5403. DOI: 10.1021/acs.jctc.9b00705.

(6) Woon, D. E.; Dunning, T. H., Jr. Benchmark calculations with correlated molecular wave functions. VI. Second row A2 and first row/second row AB diatomic molecules. *The Journal of Chemical Physics* **1994**, *101* (10), 8877-8893. DOI: 10.1063/1.468080 (acccessed 10/27/2023).
